# A scoping review of the reporting quality of reviews of commercially and publicly available mobile health apps

**DOI:** 10.1093/jamiaopen/ooae159

**Published:** 2025-01-13

**Authors:** Norina Gasteiger, Gill Norman, Rebecca Grainger, Sabine N van der Veer, Lisa McGarrigle, Debra Jones, Charlotte Eost-Telling, Amy Vercell, Claire R Ford, Syed Mustafa Ali, Kate Law, Qimeng Zhao, Matthew Byerly, Chunhu Shi, Alan Davies, Alex Hall, Dawn Dowding

**Affiliations:** Division of Nursing, Midwifery and Social Work, The University of Manchester, Manchester M13 9PL, United Kingdom; National Institute for Health and Care Research Applied Research Collaboration Greater Manchester, The University of Manchester, Manchester M13 9PL, United Kingdom; NIHR Innovation Observatory, Population Health Sciences Institute, Newcastle University, Newcastle NE1 7RU, United Kingdom; Department of Medicine, University of Otago Wellington, Wellington 6242, New Zealand; National Institute for Health and Care Research Applied Research Collaboration Greater Manchester, The University of Manchester, Manchester M13 9PL, United Kingdom; Centre for Health Informatics, Division of Informatics, Imaging and Data Sciences, Manchester Academic Health Science Centre, The University of Manchester, Manchester M13 9PL, United Kingdom; Division of Nursing, Midwifery and Social Work, The University of Manchester, Manchester M13 9PL, United Kingdom; National Institute for Health and Care Research (NIHR) Policy Research Unit in Healthy Ageing, School of Health Sciences, Faculty of Biology, Medicine and Health, The University of Manchester, Manchester M13 9PL, United Kingdom; Manchester Academic Health Science Centre, Manchester M13 9NQ, United Kingdom; Division of Nursing, Midwifery and Social Work, The University of Manchester, Manchester M13 9PL, United Kingdom; Division of Nursing, Midwifery and Social Work, The University of Manchester, Manchester M13 9PL, United Kingdom; National Institute for Health and Care Research Applied Research Collaboration Greater Manchester, The University of Manchester, Manchester M13 9PL, United Kingdom; The Christie NHS Foundation Trust, Manchester M20 4BX, United Kingdom; Division of Nursing, Midwifery and Social Work, The University of Manchester, Manchester M13 9PL, United Kingdom; National Institute for Health and Care Research Applied Research Collaboration Greater Manchester, The University of Manchester, Manchester M13 9PL, United Kingdom; Centre for Health Informatics, Division of Informatics, Imaging and Data Sciences, Manchester Academic Health Science Centre, The University of Manchester, Manchester M13 9PL, United Kingdom; The Christie NHS Foundation Trust, Manchester M20 4BX, United Kingdom; Division of Nursing, Midwifery and Social Work, The University of Manchester, Manchester M13 9PL, United Kingdom; The University of Kansas School of Medicine-Wichita, Wichita, KS 67214, United States; Division of Nursing, Midwifery and Social Work, The University of Manchester, Manchester M13 9PL, United Kingdom; National Institute for Health and Care Research Applied Research Collaboration Greater Manchester, The University of Manchester, Manchester M13 9PL, United Kingdom; Centre for Health Informatics, Division of Informatics, Imaging and Data Sciences, Manchester Academic Health Science Centre, The University of Manchester, Manchester M13 9PL, United Kingdom; Division of Nursing, Midwifery and Social Work, The University of Manchester, Manchester M13 9PL, United Kingdom; Division of Nursing, Midwifery and Social Work, The University of Manchester, Manchester M13 9PL, United Kingdom; National Institute for Health and Care Research Applied Research Collaboration Greater Manchester, The University of Manchester, Manchester M13 9PL, United Kingdom

**Keywords:** app review, mHealth, smartphone, research methods, reporting, scoping review

## Abstract

**Objectives:**

There is no guidance to support the reporting of systematic reviews of mobile health (mhealth) apps (app reviews), so authors attempt to use/modify the Preferred Reporting Items for Systematic Reviews and Meta-Analyses (PRISMA). There is a need for reporting guidance, building on PRISMA where appropriate, tailored to app reviews. The objectives were to describe the reporting quality of published mHealth app reviews, identify the need for, and develop potential candidate items for a reporting guideline.

**Materials and Methods:**

A scoping review following the Joanna Briggs Institute and Arksey and O’Malley approaches. App reviews were identified in January 2024 from SCOPUS, CINAHL, AMED, EMBASE, Medline, PsycINFO, ACM Digital Library, snowballing reference lists, and forward citation searches. Data were extracted into Excel and analyzed using descriptive statistics and content synthesis, using PRISMA items as a framework.

**Results:**

One hundred and seventy-one app reviews were identified, published from 2013 to 2024. Protocols were developed for 11% of the reviews, and only 52% reported the geographical location of the app markets. Few reported the duplicate removal process (12%), device and operating system used (30%), or made clear recommendations for the best-rated apps (18%). Nineteen PRISMA items were not reported by most (>85%) reviews, and 4 were modified by >30% of the reviews. Involvement of patient/public contributors (4%) or other stakeholders (11%) was infrequent. Overall, 34 candidate items and 10 subitems were identified to be considered for a new guideline.

**Discussion and Conclusion:**

App reviews were inconsistently reported, and many PRISMA items were not deemed relevant. Consensus work is needed to revise and prioritize the candidate items for a reporting guideline for systematic app reviews.

## Background and significance

Systematic reviews of mobile health (mHealth) applications (apps) are a relatively new method of reviewing the health-related app market, often focusing on evaluating the availability, quality, and functionality of commercially and publicly available products. The potential implications of these reviews are 3-fold: they can inform health care decision-makers, practitioners, patients and the general public seeking high-quality apps; help identify gaps in the field to guide researchers and industry in developing new products; and inform evaluation research.^1^ Although mHealth app reviews are recognized as a valid systematic review method in health care,^2^ there is no consensus on their conduct and reporting. This contrasts with the widely known Preferred Reporting Items for Systematic Reviews and Meta-Analyses (PRISMA) reporting guidelines^3^ and associated extensions available to guide the conduct and reporting of reviews of the academic literature.

Reporting guidelines may lead to better transparency and standardization of research publications, while their absence can contribute to research waste and mislead research consumers if important information is omitted. In 2020, a review of 26 mHealth app reviews highlighted concerns with their reporting.^4^ For example, in 20 of the reviews, it was unclear if screening apps for inclusion in the review was conducted independently and 20 reviews did not include clinical recommendations. The lack of standardization in reporting this information highlights uncertainty about the robustness of the methods, in turn compromising usefulness, trust in findings, and the ability to make informed clinical decisions regarding the use of apps.

Currently, many mHealth app review authors attempt to use the PRISMA guidelines, even though the conduct of app reviews differs from that of a traditional evidence review. For example, searches take place on commercial app markets (eg, the Google Play or Apple App Store) instead of stable bibliographic databases, screening is often performed on Microsoft Excel (rather than a tool designed specifically for this task such as Rayyan^5^ or Covidence^6^) and the whole process must be conducted in a relatively rapid manner as apps may become unavailable, be updated or new apps may be released, which means results are not reproducible.^7^ App review authors have highlighted that PRISMA is not fit for purpose for mHealth app reviews.^4^^,^^8–10^ In their review of exercise apps, Soto-Bagaria et al.^8^ acknowledge that they attempted to use the PRISMA guidelines but it is not clear whether these guidelines are a valid framework, and that no gold standard for the conduct and reporting of mHealth app reviews currently exists. Similarly, Robinson et al.^9^ state that some items in the PRISMA checklist were not relevant to their review of asthma apps, including 12 items related to risk of bias, effect measures for each outcome, synthesis methods, addressing certainty, reporting biases, and certainty of evidence. There has been a call to prioritize the standardization of the systematic mHealth app review method and reporting, including using more systematic and rigid protocols explicitly developed for mHealth app reviews.^4^^,^^11^^,^^12^

Some attempts have been made to create app review reporting guidelines. Marshall et al.^10^ developed the Protocol for App Store Systematic Reviews guidance by simply amending some of the PRISMA^3^ and AMSTAR 2 items, the latter of which is a critical appraisal tool for systematic reviews that include randomized or nonrandomized studies.^13^ However, the authors did not systematically identify candidate items to include in the reporting guidelines. Their work was also not consensus-driven nor invited input from stakeholders, such as app review authors, patients, the public, software developers, and health care decision-makers and providers. There is no description about how the guideline was developed nor registration as a formal reporting guideline with the EQUATOR Network (an international reporting guideline database).^14^ As a result, the relevance of many items remains uncertain. When assessing the evidence for digital interventions, the new PICOTS-ComTec framework would be more suitable as this also considers the setting, communication, technology, and context.^15^ However, for systematic mHealth app reviews which deal with the products/interventions directly, a more appropriate and consensus-driven guideline is needed.

This scoping review is part of a broader project aiming to standardize the mHealth app review method and develop the Consensus for APP Review Reporting Items (CAPPRRI) reporting guidance. It responds directly to the call for the standardization of the conduct and reporting of systematic mHealth app reviews^4^^,^^11^^,^^12^ and contrasts previous attempts to develop a reporting guideline (eg, Marshall et al.^10^) by employing a more systematic, transparent, and consensus-driven approach. To date, this has involved establishing the foundations for systematizing the app review method, publishing an article on the methodological considerations.^7^ This has influenced the conduct of new reviews^16^^,^^17^ and was recommended by app review authors as a systematic approach that should be embraced.^11^ The Moher et al.^18^ guidance for developers of health research reporting guidelines is being followed to develop the CAPPRRI guidance, including identifying the need for a new guideline and reviewing the literature to collate evidence on the quality of reporting of published articles.

### Aim

The aims of this review are 3-fold. Firstly, to describe the reporting quality of published app reviews; secondly to identify the need for a new reporting guideline by exploring how published reviews have aligned to, deviated from, and modified the PRISMA 2020 items; and thirdly, to develop candidate items for inclusion in a potential new mHealth app review guideline.

## Methods

### Approach and registration

The Joanna Briggs Institute strategy for scoping reviews^19^ and the 5-step process for conducting scoping reviews (Arksey and O’Malley^20^) have been used to guide the scoping review. The review is reported using the PRISMA-ScR extension guidelines for scoping reviews^21^ (see File S1).

The protocol, registered in Open Science Framework (OSF) (https://osf.io/5ahjx) and published in BMJ Open^1^ provides a detailed description of this scoping review. A teaching and learning librarian gave input on the search strategy. A public and patient involvement and engagement (PPIE) group also gave input on the protocol, suggesting additional items to extract and ideas for presenting and disseminating findings. Two amendments were made to the protocol during the review; due to the number of published reviews, eligibility criteria were adjusted to only include mHealth app reviews that mentioned PRISMA (or any PRISMA extension) in the methods and where authors downloaded the apps for review.

### Search and screening

A 3-stage approach was used to identify eligible mHealth app reviews. In January 2024, 1 author (N.G.) conducted database searches of SCOPUS, CINAHL Plus (via EBSCO), AMED (via Ovid), EMBASE (via Ovid), Medline (via Ovid), APA PsycINFO, and ACM Digital Library (details in File S1). Limits were placed on the publication date (January 1, 2007) as the first iPhone model (and first smartphone) was released on June 29, 2007. The eligibility criteria^1^ are presented in Table 1 and were developed using the study, data, methods, and outcome (SDMO) acronym.^23^

**Table 1.** Eligibility criteria.

SDMO acronym	Inclusion criteria	Exclusion criteria	No limits
Types of study	Reviews of commercial and publicly available mobile apps *Must be focused on smartphone (mobile) apps* *Can be identified as systematic, scoping or without a specific approach named* *Some app reviews may be combined with other literature reviews or reviews of other apps. These will only be included if detail is reported separately on the smartphone app review methods and results* *Reviews including other technology (eg, iPads, digital assistants, virtual reality headsets or smartwatches) will only be included if the focus is on smartphone apps and the other technology is used only to operationalize some of the functions* English language Published on or after January 1, 2007	Literature reviews Reviews of other technology or apps (eg, websites, computer apps, iPad apps) Full text not available *Exclude abstracts and documents where there is insufficient information or the full text is not available* Not in English Published before January 1, 2007	Document type *Any document type will be included, if there is a full-text available so that enough information can be extracted (eg, full-length conference papers, journal articles, book chapters).* Smartphone device Operating system requirements App markets Geography (location)
Types of data	Health focus *Must be focused on a health topic, whereby the apps are marketed for physical or mental health or general well-being. This may include (but is not limited to) apps that educate, empower, or inform users on a health topic (eg, genetics), self-monitor/manage or change health behaviors (eg, sleep, nutrition, exercise or smoking cessation), or are used for social support or in health systems or by patients, administrators and health and care workers or decision-makers (eg, screening, diagnosis, triage, appointment-booking, remote monitoring, decision-making, training, and treatment)*	Not focused on health topics	Health topic *Apps can be for any health topic.* Intended users of apps *Apps can be for any stakeholder, including patients, the public, health professionals and the health system.*
Types of methods	Authors must have downloaded the apps for review^a^ Authors have named or cited PRISMA (or any extension) in the Methods.^a^ *This includes for reporting, developing the search strategy, or presenting the flow diagram. Extensions could include but are not limited to PRISMA-Scr, PRISMA-P, or the updated PRISMA (2020)* Any method (and measure) of evaluating apps can be included, such as using validated measures (eg, MARS^22^), synthesizing content presented within the app or user ratings and reviews on app markets	Authors did not download the apps for review (eg, only summarized their names and descriptions in the app stores)^a^ Authors did not name or cite PRISMA^a^	App evaluation measures and methods.
Types of outcomes	Any outcome, including those related to evaluating app quality, functionality, privacy and security, accessibility, or efficacy. This would also include app reviews that simply focus on identifying which apps were available, summarized their content or describing the extent to which they adhere to best clinical practice/guidelines	None	Any outcomes.

Abbreviation: SDMO, study, data, methods, and outcome.

a Criteria modified (added) after protocol development and publication, due to feasibility.

Results were imported into Rayyan^5^ for manual deduplication and screening. The deduplication process was completed by the lead author (N.G.). This process consisted of Rayyan detecting potential duplicates and the researcher manually checking each record’s information (eg, title, publication date, authors, journal, abstract, and DOI). Once resolved, duplicates were filtered from the records that required screening. The authors involved in the screening process (N.G., S.M.A., A.V., G.N., C.R.F., C.E.-T., D.J., and L.M.) piloted the screening procedure (ie, eligibility criteria and labeling process on Rayyan) on 50 records with an overall agreement rate of 80% (40/50). Next, 5 pairs of 2 authors independently screened the titles/abstracts and met to discuss disagreements. The third step included the full-text screening in the same pairs, with discussions to resolve disagreements. A third author (N.G. or G.N.) was available to help reach a consensus. All screening was blinded.

Five authors (N.G., S.M.A., L.M., C.E.-T., and D.J.) then independently snowballed additional reviews from reference lists of eligible articles (identified from the database searches). A forward citation approach was also used to identify app reviews that had cited earlier published work. During the full-text screening stage, app reviews that were excluded due to not being in English were recorded (see File S1) to enable others to identify these papers for subsequent reviews.

### Data extraction

Data extraction was informed by the items extracted in Grainger et al.,^4^ the TECH framework^7^ and discussions with the PPIE group and the researchers involved in data extraction (see File S1). To evaluate reporting quality, we extracted information about the title, topic, setting, target users, protocol, review questions, app store search and screening, app evaluation, findings, and stakeholder engagement. To identify the need for a new guideline, each review was assessed for its reporting of PRISMA 2020 items^3^ and if applicable, we summarized any modifications that had been made.

The extraction sheets were developed iteratively, with amendments made during the piloting process with 4 reviews. The authors involved in extraction then piloted 6 reviews with an average team agreement rate of 81%, after which disagreements were discussed. The remaining 161 reviews were extracted by 9 researchers (N.G., S.M.A., A.V., C.R.F., C.E.-T., D.J., L.M., Q.Z., and K.L.), with 10% (*n* = 17) of extractions reviewed by a second author.

### Collating, summarizing, and presenting the results

Where possible, data were analyzed in Excel and reported as frequencies to indicate the extent to which items were reported. The data extracted to evaluate the reporting quality informed the development of candidate items. A content synthesis approach was used to summarize how the PRISMA 2020 items had been modified. This led to the development of some candidate items that draw on the basis of PRISMA but are more relevant to app reviews. The items were then combined into a list which was sense-checked by the research team.

## Results

**Figure 1.**
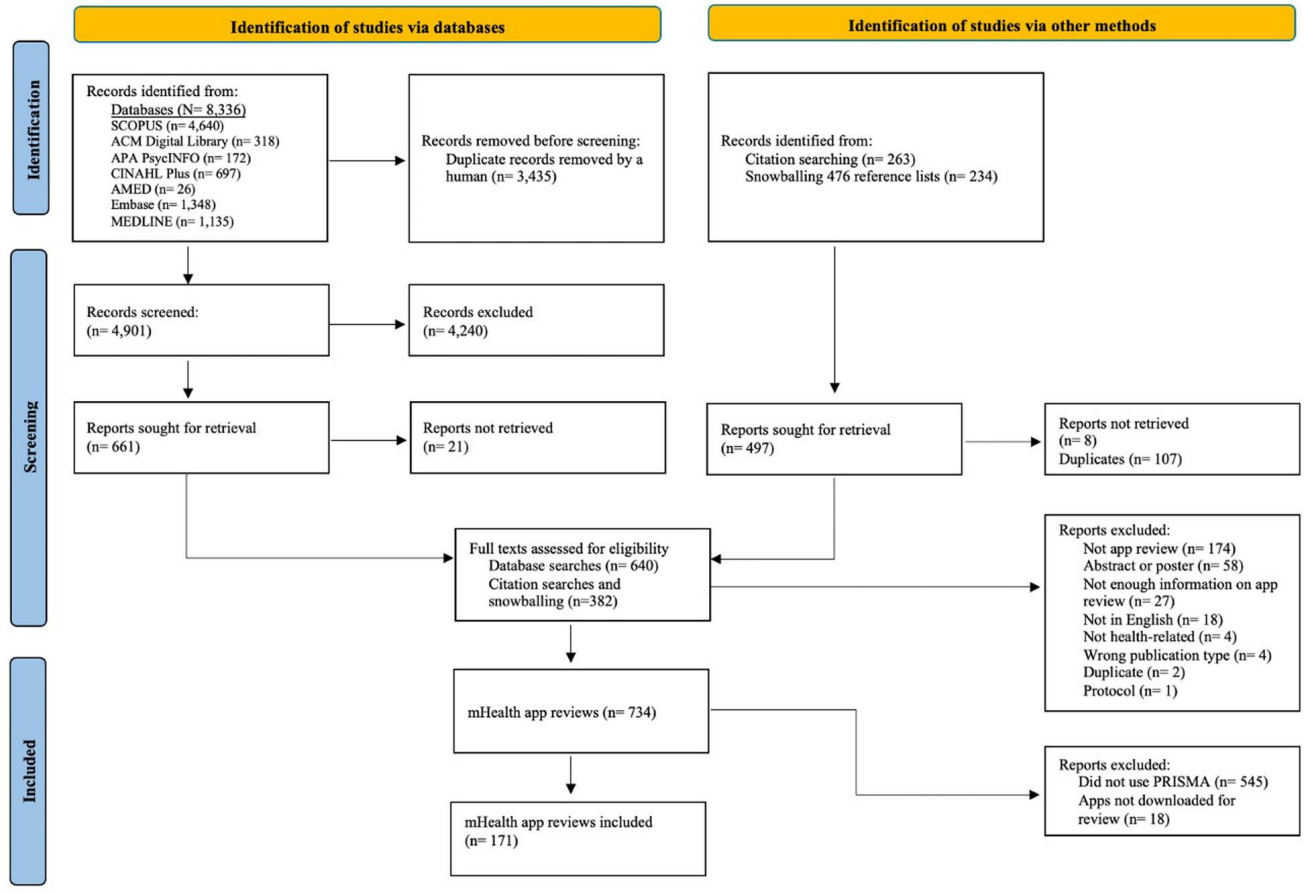
Flow diagram depicting the search and screening process.

The database searches yielded 8336 results, and a further 497 records were identified via citation searching and snowballing. The overall team agreement rate for the title/abstract screening was 94% (92%-95% agreement within the pairs). A total of 1022 full texts were considered for inclusion. The agreement rate at the full-text stage was 93% (ranging from 90% to 97%). The most common reasons for exclusion were not being an app review (*n* = 174), being an abstract or poster (*n* = 58) and not providing enough information (*n* = 27). There were 734 app reviews, but 545 did not use, cite, or name PRISMA and 18 did not download the apps for review. A total of 171 mHealth app reviews were included in this scoping review (Figure 1).

### Characteristics of the included reviews

The reviews were published from 2013 to 2024, with 82% published in the last 4 years (2020-2024). Most of the included app reviews (95%, *n* = 162) were published as journal articles with the remainder being conference papers (*n* = 4), preprints (*n* = 3), or a thesis/dissertation (*n* = 2).

### Evaluating the reporting quality

#### Title and method

Although 128 (75%) reviews named a method in the title, there was great variation in how this was presented, with only 4% (*n* = 7) solely being named app reviews and more than 1 method sometimes used. Fifty-nine percent (*n* = 101) were named systematic, but this was often coupled with keywords related to screening, evaluation, assessment, search, or app review (eg, systematic search in app stores). Eight percent (*n* = 13) were named scoping reviews and 10% (*n* = 17) were named with other descriptors such as review of content, quality or functionalities, rapid review or descriptive review.

The majority (77%, *n* = 132) were standalone reviews of the mHealth app market/s. Only 23% (*n* = 39) were combined with other methods, such as systematic, scoping or narrative reviews of the literature, or primary data collection (eg, interviews with developers or surveys asking participants which apps they used).

#### Topic, setting, and target users

**Figure 2.**
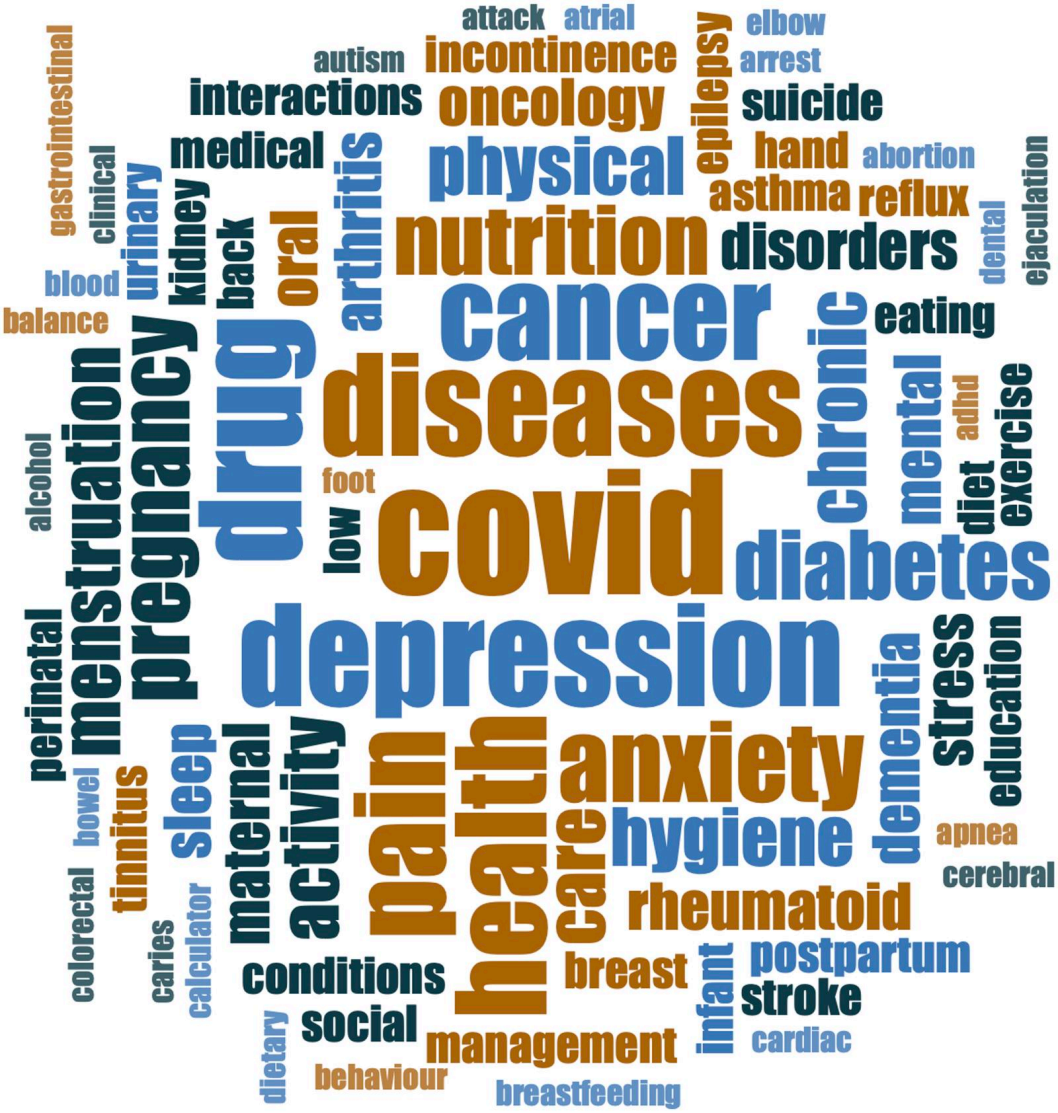
Word cloud representing the health topics explored in the mHealth app reviews.

**Figure 3.**
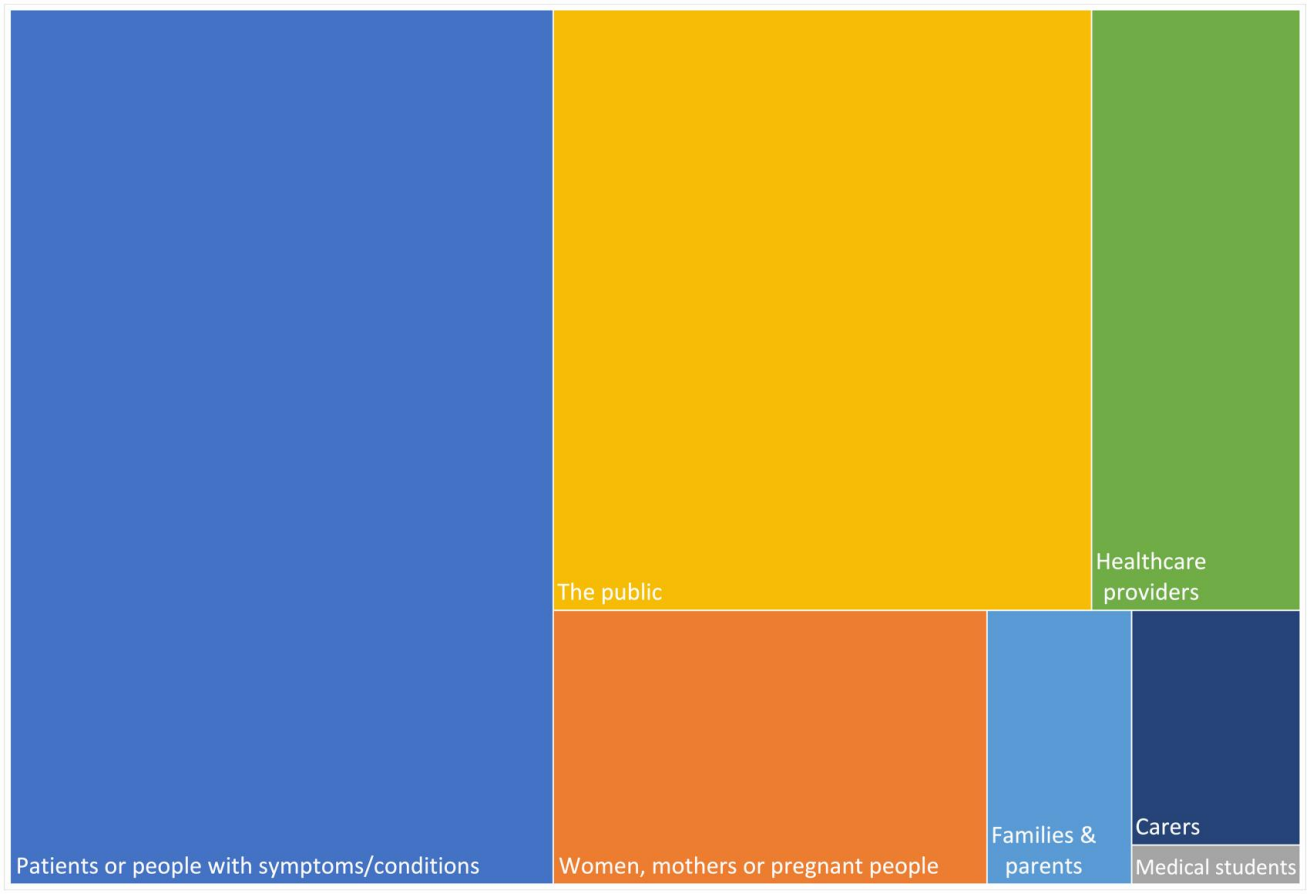
Treemap showing the expected target users of the reviewed apps.

The 171 app reviews focused on a range of health topics (Figure 2) with the largest proportion of target users being patients or members of the public (Figure 3). Most reviews (91%, *n* = 156) defined the health care setting. This was primarily the community (*n* = 136, eg, self-management or improving health literacy) but also included health care settings such as primary care, acute care or emergency medicine, secondary care (eg, oncology, hospitals, neonatal intensive care unit), or long-term care. A total of 11 421 apps were reviewed, with an average of 67 apps reviewed per publication.

#### Protocol and registration

Only 11% (*n* = 19) of reviews had developed a protocol for the app review, of which most (84%, *n* = 16) were published on PROSPERO. Of the 16 published on PROSPERO, 5 were combined with evidence reviews. Two were published on OSF and 1 mentioned a protocol but did not report the place of publication.

#### Development and framing of the review questions

Few reviews reported using a standardized approach to framing the research question or search—with 7 using PICO (patient/population/problem, intervention, comparison/control, outcome). One used the PCC (population, concept, context) acronym.

We applied the TECH (Target user, Evaluation focus, Connectedness, Health domain) components to the research questions.^7^ Half (51%, *n* = 87) included 3 components, while only 2% (*n* = 3) included all 4. The Health domain was the most commonly reported (*n* = 168), followed by the Evaluation focus (*n* = 156) and the Target user (*n* = 100). Connectedness was the least commonly reported (*n* = 4).

#### App store search and screening method

Searches: Most (96%, *n* = 164) reported keywords used in the app market searches and the month and year the search was conducted (85%, *n* = 146). Over half (52%, *n* = 89) of the reviews explicitly reported the geographical location of the app markets they searched. Of these, 2 conducted worldwide searches and 2 searched the European market. Ten searched more than 1 country. Details on country distribution are in File S1.

Screening: Most (83%, *n* = 141) also presented limits on the inclusion of apps based on relevant criteria such as lite/full versions, paid/free apps, and language, with 57% of the reviews (*n* = 97) reporting the number and independence of people screening/identifying the apps. There were 1-7 people involved in the screening (mode: 2; average 2.4; SD: 1), including helping to resolve disagreements.

The duplicate removal process was also not reported or unclear in 88% (*n* = 150). Only 12% (*n* = 20) reported how the data were managed (eg, where the duplicates were viewed—app store pages, screen grabs, Excel) and what information helped to determine if apps were duplicates (eg, developer, version numbers). For 165 reviews, multiple app platforms were searched. Of these 24% (*n* = 40) clearly reported whether (and which) apps that appeared on multiple platforms were included.

#### Evaluation of the apps

Reviewers and devices: For the review process, 64% of the reviews (*n* = 109) clearly reported the number and independence of people reviewing the apps with 1-22 people involved in the review process (mode: 2; average 3; SD: 2.9). However, smaller teams were often formed, for example, in a team of 18 reviewers, each app was only evaluated by 2 people. Only 30% (*n* = 52) reported the device model and operating system version used when evaluating the apps. These were partially or not reported by 23% (*n* = 40) and 46% (*n* = 79), respectively.

**Figure 4.**
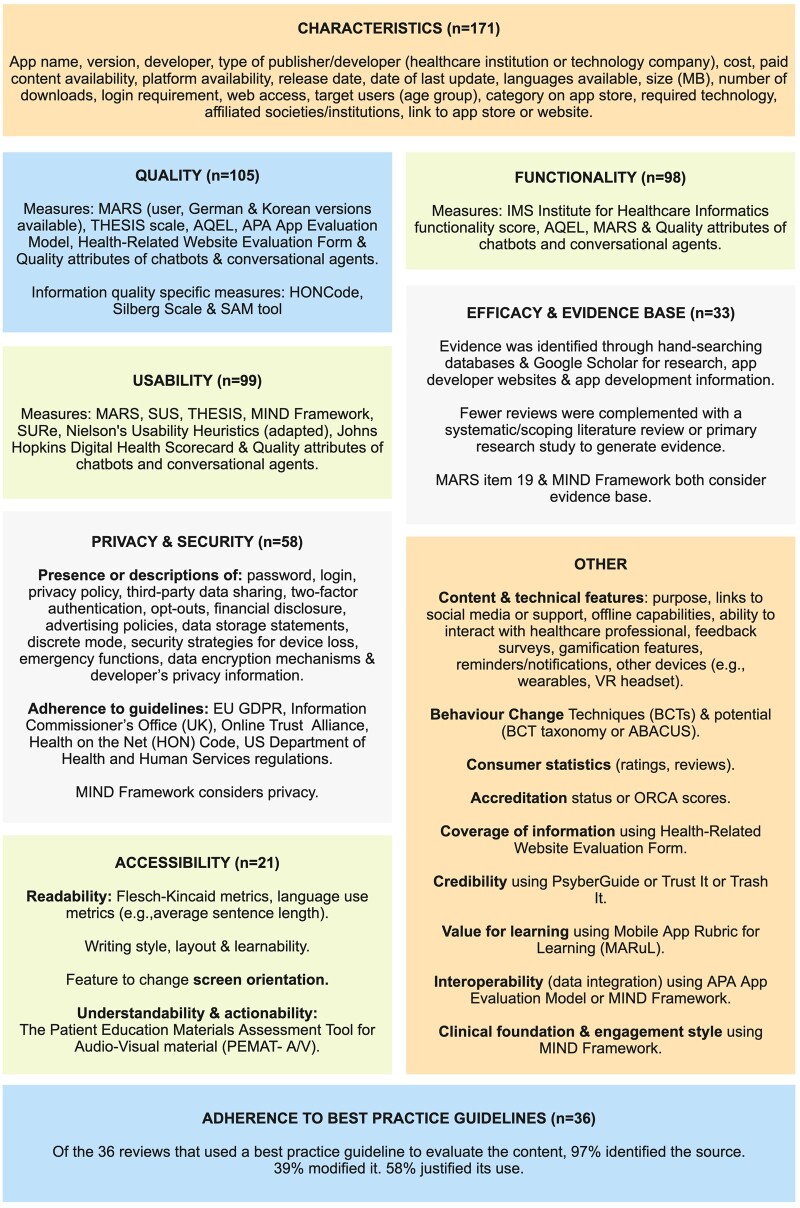
Tiles displaying the key outcomes of interest, and the measures and frameworks used.

App assessment metrics: Figure 4 summarizes the key app assessment metrics used by the reviewers. All reviews considered descriptive characteristics of the apps; 34% explored privacy/security, 21% adherence to best practice guidelines, 19% efficacy or the evidence base, and 12% accessibility. Existing (including validated) measures or frameworks were used to conduct formal quality (61% of reviews), usability (58%), and functionality (57%) assessments. The measures most used are listed in File S1.

#### Recommendations

The majority (82%, *n* = 141) of the reviews failed to make clear recommendations or conclusions for the best-rated apps. When apps were recommended, this was informed by a cross-comparison of criteria, including descriptive information, user ratings, adherence to guidelines, and evaluation measures such as scores from MARS (Mobile apps rating scale), IMS Institute of Informatics Functionality Scale and behavior change techniques (eg,^24–29^). The information was presented in the “Results” or “Discussion” section and often framed the apps as “top-rated,” “the best,” or “highest scoring.”

#### Stakeholder engagement, consultation, and the inclusion of lay summaries

Only 4% (*n* = 6) of app reviews collaborated with patients or members of the public, while 11% (*n* = 18) collaborated with health care workers, medical education leaders, epidemiologists, technology experts, app developers, and health informatics specialists. These collaborators were involved in tasks like determining the search terms and helping review/evaluate the apps. Only 1 review included a lay summary (in addition to the Abstract).^30^ However, the summary did not recommend any of the reviewed apps nor name any they reviewed.

### Identifying the need for a new reporting guideline: adherence to PRISMA

While all reviews mentioned PRISMA, 62% (*n* = 106) explicitly stated using PRISMA reporting guidelines to guide the conduct or reporting of the review. Of these, PRISMA (2009, 2020, or no date) was referred to in 85 (50%) reviews. Other reviews referred to PRISMA-P (protocols) (6%, *n* = 10) or PRISMA-ScR (scoping review) (6%, *n* = 10) extensions. The PRISMA-NMA (network meta-analyses) and the PRISMA-S (literature searches) extension were each referred to in 1 paper.

Ten reviews mentioned amending the guidelines, for example, indicating that the PRISMA flow diagram had been amended^31^ or leaving some items blank (unreported) in the checklist.^32^ Another stated that *“adjustments were needed because of the different search nature of mobile app stores”*.^33^

Flow diagrams were presented in 96% (*n* = 164) with 71% of these (*n* = 121) directly mentioning or citing PRISMA in their diagrams. However, modifications were made to accommodate the unique app market search and screening process.

#### Reporting of the PRISMA 2020 items

File S1 presents the reporting of the PRISMA Abstracts and main checklist items.

In the Abstracts checklist, 3 items were not reported by the majority (>85%) of the reviews. Two others had been modified by 30% or more of the reviews. In the main PRISMA checklist, 19 items were not reported by the majority (>85%) of the reviews. Four items had also been modified by 30% or more of the reviews. These are presented in Tables 2 and 3.

**Table 2.** PRISMA abstracts items most often not reported or modified in the included 171 systematic mHealth app reviews.

PRISMA abstracts items	Reported number (%)	Not reported number (%)	**Unclear number (%)** ^a^	Modified number (%)	Notes (if applicable)
5. Specify the methods used to assess risk of bias in the included studies	–	165 (96)^c^	–	6 (4)	Risk of bias from evaluating the apps is assessed. Interrater reliability of the app evaluation measures assessed using Cohens Kappa or Intraclass Correlation Coefficient.
7. Give the total number of included studies and participants and summarize relevant characteristics of studies	–	10 (6)	27 (16)	134 (78)^d^	Total number of reviewed apps reported and relevant characteristics summarized (eg, platform available, care focus, language)
8. Present results for main outcomes, preferably indicating the number of included studies and participants for each. If meta-analysis was done, report the summary estimate and confidence/credible interval. If comparing groups, indicate the direction of the effect (ie, which group is favored)	–	29 (17)	–	142 (83)^d^	Main outcomes presented for the app evaluations (eg, statistics for MARS scores, mean number of behavior change techniques present).
9. Provide a brief summary of the limitations of the evidence included in the review (eg, study risk of bias, inconsistency, and imprecision)	22 (13)	149 (87)^c^	–	–	N/A^b^
11. Specify the primary source of funding for the review	120 (70)	51 (30)	–	–	Considered “reported” if anywhere in the publication, due to individual formatting requirements of publications.
12. Provide the register name and registration number	12 (7)	159 (93)^c^	–	–	N/A

Abbreviation: PRISMA, Preferred Reporting Items for Systematic Reviews and Meta-Analyses.

a Unclear refers to items that are partially reported.

b Not applicable.

c Items that were not reported by >85% of the reviews.

d Items that had been modified by >30% of the reviews.

**Table 3.** PRISMA items most often not reported or modified in the included 171 systematic mHealth app reviews.

PRISMA items	Reported number (%)	Not reported number (%)	**Unclear number (%)** ^a^	Modified number (%)	Notes (if applicable)
10b. List and define all other variables for which data were sought (eg, participant and intervention characteristics, funding sources). Describe any assumptions made about any missing or unclear information	–	171 (100)^c^	–	–	N/A^b^
11. Specify the methods used to assess risk of bias in the included studies, including details of the tool(s) used, how many reviewers assessed each study and whether they worked independently, and if applicable, details of automation tools used in the process	–	104 (61)	–	67 (39)^d^	Modified to assess risk of bias from evaluating the apps. This included interrater reliability of the app evaluation measures (eg, MARS) using Cohens Kappa or Intraclass Correlation Coefficient.
12. Specify for each outcome the effect measure(s) (eg, risk ratio, mean difference) used in the synthesis or presentation of results	3 (2)	166 (97)^c^	–	2 (1)	N/A
13a. Describe the processes used to decide which studies were eligible for each synthesis (eg, tabulating the study intervention characteristics and comparing against the planned groups for each synthesis [item #5])	1 (1)	166 (97)^c^	–	4 (2)	N/A
13b. Describe any methods required to prepare the data for presentation or synthesis, such as handling of missing summary statistics, or data conversions	2 (1)	169 (99)^c^	–	–	N/A
13c. Describe any methods used to tabulate or visually display results of individual studies and syntheses	3 (2)	168 (98)^c^	–	–	N/A
13e. Describe any methods used to explore possible causes of heterogeneity among study results (eg, subgroup analysis, meta-regression)	–	170 (99)^c^	–	1 (1)	1 review conducted a subgroup analysis to assess whether the number of app downloads was associated with the educational content, quality or number of features.
13f. Describe any sensitivity analyses conducted to assess robustness of the synthesized results	–	171 (100)^c^	–	–	N/A
14. Describe any methods used to assess risk of bias due to missing results in a synthesis (arising from reporting biases)	–	171 (100)^c^	–	–	N/A
15. Describe any methods used to assess certainty (or confidence) in the body of evidence for an outcome	–	171 (100)^c^	–	–	N/A
16b. Cite studies that might appear to meet the inclusion criteria, but which were excluded, and explain why they were excluded	–	163 (95)^c^	–	8 (5)	5% of reviews named the apps that had been screened but were excluded. This information was often in a supplementary file.
17. Cite each included study and present its characteristics	–	29 (17)	–	142 (83)^d^	Reviewed apps were named in-text or in a supplementary file. Key characteristics presented (see Figure 4). Note: version and developer were not always named.
18. Present assessments of risk of bias for each included study	–	117 (68)	–	54 (32)^d^	32% presented interrater reliability from app evaluators (eg, Cohens Kappa, Intraclass Correlation Coefficient, Kendall’s coefficient of concordance or raw agreement [%]).
19. For all outcomes, present, for each study: (a) summary statistics for each group (where appropriate) and (b) an effect estimate and its precision (eg, confidence/credible interval), ideally using structured tables or plots	2 (1)	169 (99)^c^	–	–	N/A
20a. For each synthesis, briefly summarize the characteristics and risk of bias among contributing studies	–	171 (100)^c^	–	–	N/A
20c. Present results of all investigations of possible causes of heterogeneity among study results		168 (98)^c^	–	3 (2)	Example of modification includes subgroup analysis to assess whether the number of app downloads was associated with the educational content, quality or number of features.
20d. Present results of all sensitivity analyses conducted to assess the robustness of the synthesized results	1 (1)	170 (99)^c^	–	–	N/A
21. Present assessments of risk of bias due to missing results (arising from reporting biases) for each synthesis assessed	–	171 (100)^c^	–	–	N/A
22. Present assessments of certainty (or confidence) in the body of evidence for each outcome assessed	–	171 (100)^c^	–	–	N/A
23b. Discuss any limitations of the evidence included in the review	–	50 (29)	–	121 (71)^d^	Discussed limitations of the apps reviewed.
24a. Provide registration information for the review, including register name and registration number, or state that the review was not registered	18 (11)	152 (89)^c^	1 (1)	–	1 review provided registration details for the literature review component, not the app review.
24b. Indicate where the review protocol can be accessed, or state that a protocol was not prepared	17 (10)	153 (89)^c^	1 (1)	–	N/A
24c. Describe and explain any amendments to information provided at registration or in the protocol	–	171 (100)^c^	–	–	N/A

Abbreviation: PRISMA, Preferred Reporting Items for Systematic Reviews and Meta-Analyses.

a Unclear refers to items that are partially reported.

b Not applicable.

c Items that were not reported by >85% of the reviews.

d Items that had been modified by >30% of the reviews.

### Candidate items for inclusion in a future guideline

The list of 34 candidate items (and 10 subitems) for inclusion in a future guideline is presented in Table 4. Eight items were informed by the evaluation of the reporting quality and cover the lay summary, protocol and registration, stakeholder engagement, app screening and evaluation, and making recommendations. Twenty items were informed by PRISMA modifications and reporting. These cover the abstract, introduction (ie, rationale), risk of bias, data analysis, results, discussion, and other information (eg, sources of support and conflicts of interest). Six additional items were informed by both the evaluation of the reporting quality and PRISMA modifications and reporting. These cover the title, abstract, aim/objective/research question, eligibility criteria, search strategy, and the data extraction method.

**Table 4.** Candidate items to be considered for inclusion in a future guideline.

Number	Location	Items
1^a^^,^^b^	Title	Identify the review as a systematic app review
2^b^	Abstract	State the reviews’ objective/s, aim or review/research question
3^b^		State the eligibility criteria for the included apps
4^a^^,^^b^		Specify the app stores (and if relevant, databases) used to identify and retrieve apps, the geographical location (country) and date of searches
5^b^		Name the method/s used to analyze or synthesize the data generated for the app evaluation (eg, descriptive statistics, content synthesis)
6^b^		Specify the total number of apps reviewed and summarize relevant characteristics (eg, platform available, care focus, language)
7^b^		Present the results for the main outcomes of the app evaluation (eg, mean MARS scores, and mean number of behavior change techniques present)
8^b^		Interpret the results and outline key implications
9^a^	Lay summary	Present a summary written in lay language (or in the language of the target audience). If relevant to the aim of the review, name the best rated apps, detailing the criteria used to make this judgement
10^b^	Introduction	Describe the rationale for the review in the context of existing knowledge
11^a^^,b^	Aim/research question/objective	State the reviews’ objective/s, aim or review/research question Report whether (and how) a framework was used to frame the review question/aim/objectives and determine the eligibility criteria. The TECH framework is recommended as it was designed for app reviews *Example: This review aims to identify UK patient-facing (T) cancer (H) apps with the ability to input ePROMs, and to explore their purpose, functionality, quality, (E) and ability to integrate with EHRs (C)*
12^a^	Methods	Protocol and registration Report whether a protocol was developed, where it is available (name of register and registration ID, URL, or citation). It is recommended that protocols are published in OSF. Outline any amendments made from the protocol. If the app review was not registered, state this
13^a^		Stakeholder engagement and consultation Explain any stakeholder engagement and consultation (eg, who was included, in what capacity, what training they received and what insight this bought to the review)
14^a^^,^^b^		Eligibility criteria Specify the inclusion and exclusion criteria for the review, considering aspects such as the inclusion of lite/full versions, paid/free apps and language
15^a^^,^^b^		Searches Present the full search strategies for all sources used to identify and retrieve apps, including the app stores/platforms (and if relevant, academic databases) searched, the date of searches, the geographical location (country) of the app markets/platforms, any keywords, filters and limits used. State whether searches were conducted manually or using a web crawler
16^a^		Screening Report the following detail for the screening process: Limits on the inclusion of apps (eg, lite/full versions, paid/free apps and language) The number and independence of people involved in screening the apps for inclusion Details of any tools/software used Duplicates removal process How duplicates were viewed/identified (eg, app store pages, screen grabs, Excel) What information was used to determine if apps were duplicates (eg, developer, version numbers). For multiplatform apps (ie, apps that are available in multiple platforms), state which app was included For example, *For apps that appeared on both Google Play and the Apple App store, we included both in the review*
17^a^		Evaluating the apps: reviewers Specify the number and independence of people involved in reviewing/evaluating the apps
18^a^		Evaluating the apps: devices State the device model and correlated version of the operating system used when evaluating the apps
19^a^^,^^b^		Evaluating the apps: methods used to extract data Outline the methods used to collect data from the apps, including: how many reviewers collected data from each app whether reviewers worked independently details of any tools/software used
20^a^		Evaluating the apps: assessment metrics Describe all assessment metrics, such as descriptive characteristic data collected from the apps and validated and bespoke evaluation measures used. Where applicable, cite these, and justify any modifications If considering efficacy, state whether there is evidence of specific benefit from academic institutions, end user feedback, or research studies
21^b^		Risk of bias Assess the risk of bias related to evaluating the apps. This may include calculating interrater reliability of the app evaluation measures (eg, MARS or IMS Institute of Informatics Functionality Scale) using Cohen’s kappa or intraclass correlation coefficient
22^b^		Data analysis Describe any methods used to synthesize/analyze the data and justify the choice
23^b^	Results	Describe the results of the app market search and screening process, stating how many apps were considered at each stage. Complement this with a flow diagram
24^b^		Name the reviewed apps (and their version number and developer) in-text or in the supplementary file and present their key characteristics (eg, main purpose, size (MB), platform available, and number of downloads)
25^b^		If relevant, present results of interrater reliability calculations for the app evaluations (eg, Cohen’s kappa, intraclass correlation coefficient, Kendall’s coefficient of concordance or raw agreement [%])
26^b^		Provide results of all syntheses/analyses conducted, including descriptive and inferential statistics (if relevant)
27^a^		Recommendations If relevant to the aim of the review, name the best rated apps, detailing the criteria used to make this judgement. If relevant, state how evidence of specific benefit from academic institutions, end user feedback, or research studies has informed the recommendation
28^b^	Discussion	Interpret the results in the context of existing evidence
29^b^		Discuss limitations of the apps included in the review
30^b^		Discuss limitations related to the review methods (eg, search/screening of app databases, evaluation of the apps or analysis)
31^b^		Discuss implications of the results for practice (including technology development, use or implementation), policy, and future research (including app review methods)
32^b^	Other	Acknowledge sources of support (financial or otherwise) for the conduct and publishing of the review
33^b^		Declare any relevant conflicts of interest
34^b^		If relevant, explain where any of the app review resources can be accessed (eg, data collection forms, data from the app evaluation measures or any other material)

For app reviews combined with reviews of the evidence or primary research other relevant guidelines should be drawn on.

Abbreviations: OSF, Open Science Framework; MARS, Mobile App Rating Scale; PRISMA, Preferred Reporting Items for Systematic Reviews and Meta-Analyses.

a Items informed by the evaluation of reporting quality.

b Items informed by the adherence and modifications to PRISMA reporting guidelines.

While these items can be used by authors in the interim, they are subject to further research to reach a consensus, prioritize them and generate a more manageable reporting list. Elaborations may also need to be developed.

## Discussion

This scoping review identified and examined the reporting quality of 171 mHealth app reviews published from 2013 to 2024. While the app review methods are frequently reported, approaches vary; protocols were only developed for some reviews (and registered in a small minority), and the geographical location, number and independence of the people screening and reviewing the apps, duplicate removal process, device model, and operating system were not consistently or clearly reported by many of the reviews. Very few of the reviews involved PPIE contributors or other stakeholders. Furthermore, the majority did not make clear recommendations or conclusions about the most suitable apps for use by target users.

The need for a new, systematic guideline designed explicitly for reporting mHealth app reviews was evident as there is a currently a significant lack of transparency and standardization in the conduct and reporting of app reviews. This is reflected by the fact that 45% of the main PRISMA 2020 checklist items^3^ was not reported by more than 85% of the reviews, although all of them mentioned PRISMA. Additionally, some items were modified to suit the app review method, suggesting this guidance is not currently suited to the app review methodology. A list of candidate items to include in a potential new guideline has been developed, which also covers the reporting of stakeholder engagement and better reflects the different process of undertaking an app review.

A notable difference was uncovered regarding risk of bias for app reviews. In reviews of the evidence (eg, systematic reviews of evaluative studies), assessments of risk of bias include conducting quality appraisals, such as by using the Cochrane Risk-of-Bias tool^34^ or the Mixed Methods Appraisal Tool.^35^ Other tools such as ROBIS,^36^ the CASP Systematic Review Checklist,^37^ or AMSTAR 2^13^ are used to critically appraise systematic reviews. Equivalent tools are not available for app reviews despite the potential for quality issues analogous to bias in both the apps and the review process. For instance, bias may arise from inadequate stakeholder involvement in app development, lack of adherence to clinical guidelines, or inaccurate content. Similarly, biases can emerge during the app review process, particularly when evaluating app quality and issues such as the lack of protocols prespecifying methods are likely to contribute to this. Nevertheless, some authors have already begun calculating interrater assessments to address these biases.^26^^,^^28^^,^^29^^,^^38–41^ In the future, developing quality appraisal tools would be beneficial for quantifying bias in both mHealth apps and their review processes.

This review has also highlighted that the lack of appropriate guidance to support the conduct and reporting of app reviews may compromise their usefulness. This is due to the disconnect between the target users of the apps, intended beneficiaries or readers of the review and the data generated. For example, although many reviews explored apps for patients or the public, few explored accessibility. The apps were all for various health purposes, but few explored the adherence of the content to clinical guidelines or evidence, which would be crucial for health care professionals to consider when recommending apps. A similar finding was reported in the review by Grainger et al.,^4^ where 77% of the included reviews did not recommend apps for clinical use or report clinical efficacy. In both reviews, few included studies evaluated the app content against best practice guidelines (21% in this review, 32% in the Grainger et al.’s review).^4^ Moreover, most reviews failed to clearly identify the highest scoring apps, making it difficult for health care decision-makers, patients, and the public to choose the most suitable app.

Unsurprisingly, none of the reviews included a lay summary, as this is not currently consistently a requirement when publishing a journal paper. Some publishers have introduced lay summaries, in which researchers present a paragraph on the impact and implications of their work.^42^ However, this has only been introduced for some health journals like the *Journal of Hepatology*, where the intended audience is health care workers, not lay people.^43^ It is important to note that the general reading age across OECD countries is low, with 1 in 5 adults performing at level 1 or below and only 1 in 3 performing at level 2.^44^ If app reviews are intended to inform members of the public or patients, it would be appropriate for authors to present a summary in accessible language, in the language of the geographical location, where the app market was searched. Authors should also consider acceptable methods of disseminating information to the target users, including distribution on social media or through relevant organizations and charities, including the funder.

Only 11% of the reviews reported that a protocol had been developed, highlighting a potential for concern around methodological transparency and unnecessary duplication leading to research waste. Similar to systematic reviews of the evidence, editors play an important role in standardizing the conduct of systematic mHealth app reviews, enhancing transparency and reducing the potential for duplication by requesting authors develop protocols. Evidently, some authors (eg,^45^) have produced a protocol but have not registered it as platforms like PROSPERO do not normally register standalone reviews of apps (although 11 have previously been registered on PROSPERO). Instead, authors could register their protocols on OSF, publish them in academic journals or self-archive in institutional repositories. Editors and publishers can encourage registration by signposting to these sites.

It was promising that app review authors increasingly use validated and reliable measures to evaluate apps. Different versions and translations of the MARS^22^^,^^46^^,^^47^ had been used by 57% (*n* = 97) of authors to assess quality. This is an improvement from the review by Grainger et al.^4^ where only one-third of the articles used the MARS. At the time of the Grainger et al.’s review,^4^ MARS had only been cited 550 times,^4^^,^^22^ compared with more than 2180 citations in 2024. It is expected that the growth of the app review method will continue to lead to the uptake and development of new extensions and translations of MARS and similar validated tools.

### Strengths and limitations

The comprehensive search strategy, including database searches, bibliographic searches of eligible articles, and forward citation searches, was a strength of this review, which enabled the identification and review of relevant literature. Another strength is the diversity of the included reviews, which represented differing health topics across various populations and settings. Furthermore, they are geographically distributed, encompass both standalone and combined methodologies, and report on a wide array of metrics and measures used to evaluate mHealth apps. However, reviewing only the literature in English may limit the generalizability of the findings. In addition, the review only included studies that mentioned or cited PRISMA and downloaded mHealth apps, which might have resulted in the exclusion of other relevant app reviews, likely including those that are reported less well due to lack of any guidance. We therefore highlight the limitations in a nonrandom proportion of app reviews—those which are likely to be better reported.

## Conclusion

Systematic reviews of mHealth apps are growing in popularity and are increasingly being published. However, their reporting remains inconsistent and is often poor, despite attempts to use and modify established guidelines (eg, PRISMA 2020). The development of a new guideline explicitly designed for app reviews is needed, to further standardize the reporting. This review has influenced candidate items to inform a new guideline, which better reflect the process of conducting an app review. Work also needs to be done to promote the publication of associated protocols and lay summaries for this type of review, further promote the transparency of the method, and ensure the findings benefit target users.

## Supplementary Material

ooae159_Supplementary_Data

## Data Availability

Data sharing is not applicable to this article as no new data were collected.
