# Supplementary material for: A scoping review of the reporting quality of reviews of commercially and publicly available mobile health apps
Source: JAMIA Open. 2025 Jan 13;8(1):ooae159. doi: 10.1093/jamiaopen/ooae159 (PMC11729727; doi:10.1093/jamiaopen/ooae159)
Supplement: ooae159_Supplementary_Data [file ooae159_supplementary_data.docx]

**Supplementary file**

**Preferred Reporting Items for Systematic reviews and Meta-Analyses extension for Scoping Reviews (PRISMA-ScR) Checklist.**

| **SECTION** | **ITEM** | **PRISMA-ScR CHECKLIST ITEM** | **REPORTED?** |
| --- | --- | --- | --- |
| **TITLE** | | | |
| Title | 1 | Identify the report as a scoping review. | Yes |
| **ABSTRACT** | | | |
| Structured summary | 2 | Provide a structured summary that includes (as applicable): background, objectives, eligibility criteria, sources of evidence, charting methods, results, and conclusions that relate to the review questions and objectives. | Yes |
| **INTRODUCTION** | | | |
| Rationale | 3 | Describe the rationale for the review in the context of what is already known. Explain why the review questions/objectives lend themselves to a scoping review approach. | Yes |
| Objectives | 4 | Provide an explicit statement of the questions and objectives being addressed with reference to their key elements (e.g., population or participants, concepts, and context) or other relevant key elements used to conceptualize the review questions and/or objectives. | Yes |
| **METHODS** | | | |
| Protocol and registration | 5 | Indicate whether a review protocol exists; state if and where it can be accessed (e.g., a Web address); and if available, provide registration information, including the registration number. | Yes |
| Eligibility criteria | 6 | Specify characteristics of the sources of evidence used as eligibility criteria (e.g., years considered, language, and publication status), and provide a rationale. | Yes |
| Information sources* | 7 | Describe all information sources in the search (e.g., databases with dates of coverage and contact with authors to identify additional sources), as well as the date the most recent search was executed. | Yes |
| Search | 8 | Present the full electronic search strategy for at least 1 database, including any limits used, such that it could be repeated. | Yes |
| Selection of sources of evidence† | 9 | State the process for selecting sources of evidence (i.e., screening and eligibility) included in the scoping review. | Yes |
| Data charting process‡ | 10 | Describe the methods of charting data from the included sources of evidence (e.g., calibrated forms or forms that have been tested by the team before their use, and whether data charting was done independently or in duplicate) and any processes for obtaining and confirming data from investigators. | Yes |
| Data items | 11 | List and define all variables for which data were sought and any assumptions and simplifications made. | Yes |
| Critical appraisal of individual sources of evidence§ | 12 | If done, provide a rationale for conducting a critical appraisal of included sources of evidence; describe the methods used and how this information was used in any data synthesis (if appropriate). | N/A |
| Synthesis of results | 13 | Describe the methods of handling and summarizing the data that were charted. | Yes |
| **RESULTS** | | | |
| Selection of sources of evidence | 14 | Give numbers of sources of evidence screened, assessed for eligibility, and included in the review, with reasons for exclusions at each stage, ideally using a flow diagram. | Yes |
| Characteristics of sources of evidence | 15 | For each source of evidence, present characteristics for which data were charted and provide the citations. | Yes |
| Critical appraisal within sources of evidence | 16 | If done, present data on critical appraisal of included sources of evidence (see item 12). | N/A |
| Results of individual sources of evidence | 17 | For each included source of evidence, present the relevant data that were charted that relate to the review questions and objectives. | N/A |
| Synthesis of results | 18 | Summarize and/or present the charting results as they relate to the review questions and objectives. | Yes |
| **DISCUSSION** | | | |
| Summary of evidence | 19 | Summarize the main results (including an overview of concepts, themes, and types of evidence available), link to the review questions and objectives, and consider the relevance to key groups. | Yes |
| Limitations | 20 | Discuss the limitations of the scoping review process. | Yes |
| Conclusions | 21 | Provide a general interpretation of the results with respect to the review questions and objectives, as well as potential implications and/or next steps. | Yes |
| **FUNDING** | | | |
| Funding | 22 | Describe sources of funding for the included sources of evidence, as well as sources of funding for the scoping review. Describe the role of the funders of the scoping review. | Yes |

*From:* Tricco AC, Lillie E, Zarin W, O'Brien KK, Colquhoun H, Levac D, et al. PRISMA Extension for Scoping Reviews (PRISMAScR): Checklist and Explanation. Ann Intern Med. 2018;169:467–473. [doi: 10.7326/M18-0850](http://annals.org/aim/fullarticle/2700389/prisma-extension-scoping-reviews-prisma-scr-checklist-explanation).

**Search strategy: Keywords used.**

| **Technology** | **Review type** |
| --- | --- |
| - Mobile - Smartphone - “Cell phone” - mHealth - “mobile health” - eHealth - Tele* (to include telehealth, telemedicine and other variations) | (App or apps or application or applications) adj5^1^ review |

Note:

^1^The exact wording depends on the database and may include adj5, W/5 or N5. The ACM Digital Library does not allow the proximity function, so “app* review” will be used instead.

**Search strategy: Final search terms.**

|  | **SCOPUS** | **ACM Digital Library (The ACM Guide to Computing Literature)** | **APA PsycInfo (1806 to January Week 1 2024)** | **CINAHL Plus (EBSCO)** | **AMED (Allied and Complementary Medicine)**  **1985 to October 2023** | **Embase (1980 to 2024 Week 01)** | **Ovid MEDLINE(R) ALL (1946 to January 10, 2024)** |
| --- | --- | --- | --- | --- | --- | --- | --- |
| **Technology** | mobile | mobile | Mobile Phones/ | Mobile.mp | mobile.mp. | mobile phone/ or mobile.mp. | mobile.mp. |
|  | smartphone | smartphone | Smartphones/ | Smartphone/ | Smartphone.mp | smartphone/ | Smartphone/ |
|  | "cell phone" | "cell phone" | "cell phone".mp | “Cellular Phone”/ | "Cell phone".mp | "cell phone".mp. | Cell Phone/ |
|  | mhealth | mhealth | mhealth.mp | Mhealth.mp | mhealth.mp. | mhealth.mp. | mhealth.mp. |
|  | "mobile health" | "mobile health" | Mobile Health/ | "mobile health".mp. | "mobile health".mp. | "mobile health".mp. or mobile health application/ | "mobile health".mp. |
|  | ehealth | ehealth | ehealth.mp. | eHealth.mp. | ehealth.mp. | ehealth.mp. or telehealth/ | ehealth.mp. |
|  | tele* | tele* | tele*.mp.  Telemedicine/ | Telehealth/ | Telemedicine/ or tele*.mp. | tele*.mp. or telemedicine/ | Telemedicine/ or tele*.mp. |
| **Review type** | ( ( app OR apps OR application OR applications ) W/5 review ) ) | "app* review" | ((App or apps or application or applications) adj5 review).mp. | (App or apps or application or applications) N5 review | ((app or apps or application or applications) adj5 review).mp. | ((app or apps or application or applications) adj5 review).mp. | ((app or apps or application or applications) adj5 review).mp. |
| **Limits** | PUBYEAR > 2006 | [E-Publication Date: (01/01/2007 TO 31/12/2024)] | yr="2007 -Current" | Publication Year: 2007-2024; Publication Date: 20070101-20241231 | yr="2007 -Current" | yr="2007 -Current" | yr="2007 -Current" |

**Potential app reviews excluded at full-text screening stage due to language.**

| **Title** | **Year** | **First author** | **Journal or Conference** | **URL/DOI** | **Language** | **Identified from** |
| --- | --- | --- | --- | --- | --- | --- |
| An analysis of gamification elements in mHealth applications | 2024 | Rocha | XXII Simpósio Brasileiro de Fatores Humanos em Sistemas Computacionais | https://dl.acm.org/doi/10.1145/3638067.3638126 | Portugese | Snowballing citations |
| 정신건강 증진을 위한 기분 관리 모바일 애플리케이션의 질적 평가: Mobile Application Rating Scale의 적용. | 2023 | 안순태 | Journal of Korean Academy of Fundamentals of Nursing | 10.7739/jkafn.2022.30.2.180 | Korean | Database searches |
| Apps for tinnitus? A systematic review on quality, intervention components, and behavior change techniques | 2023 | Rinn | Bundesgesundheitsblatt - Gesundheitsforschung - Gesundheitsschutz | 10.1007/s00103-023-03805-1 | German | Database searches |
| Mobile applications for the control of anxiety and depression symptoms: search and quality evaluation | 2023 | Bunova | Profilakticheskaya Meditsina | 10.17116/profmed20232610127 | Russian | Database searches |
| Quality Evaluation of Mobile Application for Daily Mood Regulation: A Review and Analysis using the Mobile Application Rating Scale | 2023 | An | Journal of the Korean Academy of Fundamentals of Nursing | 10.7739/jkafn.2022.30.2.180 | Korean | Database searches |
| Mobile applications to improve drug adherence: Review and quality analysis | 2021 | González de León | Atencion Primaria | 10.1016/j.aprim.2021.102095 | Spanish | Database searches |
| Smartphone applications to promote physical activity in patients with rheumatoid arthritis, a research for different applications | 2021 | Rogez | Kinesitherapie | 10.1016/j.kine.2021.01.008 | French | Database searches |
| Using mobile applications and websites for the diagnosis of COVID-19 in Spain | 2021 | Guisado-Clavero | Enfermedades Infecciosas y Microbiologia Clinica | https://dx.doi.org/10.1016/j.eimc.2020.08.002 | Spanish | Database searches |
| Medical apps—possibilities for pruritus | 2020 | Schuster | Hautarzt | 10.1007/s00105-020-04603-5 | German | Database searches |
| Commercial mobile applications in the therapeutic approach to stroke: Review in main application repositories and scientific evidence | 2019 | Ortega-Martín | Revista Espanola de Salud Publica | https://www.sanidad.gob.es/biblioPublic/publicaciones/recursos_propios/resp/revista_cdrom/VOL93/O_BREVES/RS93C_201906035.pdf | Spanish | Database searches |
| Investigation of the effectiveness of mobile health applications for chronic diseases | 2019 | Spyridaki | Archives of Hellenic Medicine | http://mail.mednet.gr/archives/2019-1/73abs.html | Greek | Database searches |
| Mobile applications in alzheimer's disease. A systematic review of the literature | 2019 | Sánchez-Gutiérrez | Rehabilitacion | 10.1016/j.rh.2019.07.002 | Spanish | Database searches |
| Mobile phone applications in Parkinson's disease: A systematic review | 2019 | Linares-del Rey | Neurologia | 10.1016/j.nrl.2017.03.006 | Spanish | Database searches |
| Help from the App Store?': A Systematic Review of Depression Apps in German App Stores | 2018 | Terhorst | Verhaltenstherapie | 10.1159/000481692 | German | Database searches |
| Mobile applications related to stroke: A systematic review | 2018 | Rodríguez-Prunotto | Revista de Neurologia | 10.33588/rn.6607.2017380 | Spanish | Database searches |
| Which useful Apps are installed on my smartphone as a clinical pharmacist? | 2017 | Mille | Annales Pharmaceutiques Francaises | 10.1016/j.pharma.2017.02.002 | French | Database searches |
| Medical apps in clinical use: Mobile success | 2014 | Knipp | Deutsche Medizinische Wochenschrift | https://dx.doi.org/10.1055/s-0034-1387383 | German | Database searches |
| Aplicaciones móviles en nutrición, dietética y hábitos saludables: análisis y consecuencia de una tendencia a la alza. | 2014 | San Mauro Martín | Nutrición Hospitalaria | 10.3305/nh.2014.30.1.7398 | Spanish | Database searches |

**Data extraction items.**

| **Article characteristics** | |
| --- | --- |
| Title | - Title of the article. - Does it name a review method in the title (e.g., systematic, scoping, app review)? |
| Date | - Year of publication. |
| Journal | - Name of the journal the app review is published in. - Name and contact information of the editor-in-chief.^1^ |
| Authors | - Name and contact information of all authors.^1^ - Source of funding (if any). |
| Objective | - Aim or research question. |
| Topic and context | - The health problem being explored. - App target user. - Context: where the app is to be used, including location of care (acute, primary health care, community, long-term care, etc.) and geographical location. |
| **Protocol and registration** | |
| A priori review registration | - Was the study protocol registered and was the protocol available?^2^ |
| **Review question** | |
| Review question frameworks | - Was a framework used to write the review question (e.g., PICO)? If so, which? - Did this align with any of the TECH components? If so, which? |
| **Reporting guidelines** | |
| Reporting guidelines | - Did authors state which reporting guideline they used? If so, which? - Did the authors clearly mention amending any guidelines? |
| Alignment, deviation and modification to the PRISMA (2020) items | - For each of the PRISMA (2020) items, identify whether the information was reported as is, or how this was modified (if applicable). |
| **Search and screening** | |
| Flow charts/diagrams | - Did the authors present a flow diagram for the search and screening process? - Did the authors report a PRISMA (2020) flow chart for new or updated systematic reviews? - Were amendments made to the PRISMA (2020) flow charts? If yes, which? |
| App store search | - Were the apps store(s) searched described including the a) keywords, b) countries/location and c) a clear description of dates of the search?^2^ - Was the method for identifying and removing duplicate apps clearly described, including metadata (e.g., version numbers) used to determine if apps were duplicates?^2^ - Were the number and independence of people screening apps described?^2^ - Were limits on inclusion of apps based on other factors clearly described (lite or full version, paid or free versions, non-English)?^2^ - Where the same app featured in different app stores (multiplatform apps), was there a clear statement of which apps were included?^2^ - When apps were downloaded to phones for data extraction, were the phone model and version of the operating system clearly reported?^2^ |
| **Evaluating the apps and making recommendations** | |
| Outcomes | - Which outcomes did the review evaluate?  e.g., quality, functionality, usability, privacy, efficacy, accessibility or other |
| Quality, functionality, usability and other assessments | - Was a best practice content tool (e.g., clinical guideline) used to evaluate app content or quality? Was the source identified, were any modifications made described, and was the use of the guideline justified?^2^ - Was an app quality, functionality and/or usability evaluation undertaken using established measures and frameworks (e.g., MARS)? - Are any other tools/instruments used to evaluate app quality and/or usability, and was the source of these described?^2^ - Were security and privacy considered? If so, what aspects were included? e.g., login, password, privacy policy, access to microphone or camera, encryption or data sharing. |
| Efficacy | - Did the authors report on the efficacy of the apps? - If yes, where did they obtain this information and what methods were used? |
| Accessibility | - Was the accessibility of the apps evaluated, considering different needs of target users? |
| Recommendations | - Were any apps recommended for use in the clinical setting or by people with chronic health conditions?^2^ - Were any apps recommended overall? What informed this recommendation? |
| **Stakeholder engagement or consultation** | |
| Patient, public and expert engagement | - Were any patients or members of the public involved in the app review? - Were any other stakeholders involved or consulted (e.g., industry partners, clinicians or software developers)? If so, which? - Was a lay summary provided? If so, were any apps clearly recommended? |
| **Other** | |
| Other domains | - Any other domains reported on? If yes, what and how? |

Notes:

^1^ This information will not be published publicly as part of the review. It will be used to create a database of authors of mHealth app reviews and journals who have published them. This is required for the next step of the project- the Delphi (consensus-building) study, as mHealth experts will be participants and potential partners in this process.

^2^ These data extraction items were taken from the scoping review conducted by Grainger et al. (2020). Issues in reporting of systematic review methods in health app-focused reviews: A scoping review. *Health informatics journal*, *26*(4), 2930–2945. https://doi.org/10.1177/1460458220952917

**Names of journals that published more than one systematic mHealth app review.**

| **Journals** | **Number (%)** |
| --- | --- |
| JMIR |  |
| JMIR mHealth and uHealth | 46 (27%) |
| The Journal of Medical Internet Research | 12 (7%) |
| Oxford University Press |  |
| Journal of the American Medical Informatics Association | 2 (1%) |
| Springer Nature |  |
| Journal of Medical Systems | 4 (2%) |
| International Journal of Behavioral Nutrition and Physical Activity | 2 (1%) |
| Elsevier |  |
| International Journal of Medical Informatics | 6 (4%) |
| American Journal of Preventative Medicine | 2 (1%) |
| Computer Methods and Programs in Biomedicine | 2 (1%) |
| MDPI |  |
| Sensors | 2 (1%) |
| International Journal of Environmental Research and Public Health | 2 (1%) |
| Sage Publishing |  |
| Digital Health | 2 (1%) |
| Frontiers |  |
| Frontiers in Public Health | 2 (1%) |
| Page Press |  |
| The Archives of Italian Urology and Andrology | 2 (1%) |

Note: 76 other journals published one app review, including journals from JMIR, BMJ and the BMC series

**The 171 published reviews included.**

| **First author** | **Title** | **Year published** |
| --- | --- | --- |
| Abreu | Mobile Applications for Epilepsy: Where Are We? Where Should We Go? A Systematic Review | 2022 |
| Adnan | The real-world applications of the symptom tracking functionality available to menstrual health tracking apps | 2021 |
| Ahmed | A review of mobile chatbot apps for anxiety and depression and their self-care features | 2021 |
| Alanzi | A Review of Mobile Applications Available in the App and Google Play Stores Used During the COVID-19 Outbreak. | 2021 |
| Alfawzan | Privacy, Data Sharing, and Data Security Policies of Women's mHealth Apps: Scoping Review and Content Analysis. | 2022 |
| Alharbi | COVID-19 Mobile Apps in Saudi Arabia: Systematic Identification, Evaluation, and Features Assessment | 2022 |
| Ali | Digital manikins to self-report pain on a smartphone: A systematic review of mobile apps | 2021 |
| Almalki | Health Apps for Combating COVID-19: Descriptive Review and Taxonomy. | 2021 |
| Almoallim | Toward Research-Informed Design Implications for Interventions Limiting Smartphone Use: Functionalities Review of Digital Well-being Apps | 2022 |
| Alnooh | Identification of the Most Suitable Mobile Apps to Support Dietary Approaches to Stop Hypertension (DASH) Diet Self-Management: Systematic Search of App Stores and Content Analysis | 2023 |
| Alyami | Social anxiety apps: A systematic review and assessment of app descriptors across mobile store platforms | 2017 |
| Alzamanan | Self-management apps for people with epilepsy: Systematic analysis | 2021 |
| Amor-García | Assessing Apps for Patients with Genitourinary Tumors Using the Mobile Application Rating Scale (MARS): Systematic Search in App Stores and Content Analysis | 2020 |
| Asadollahi | Towards Effective Smartphone Pregnancy Apps: A Critical Evaluation of Quality, content and suitability in Freely Available Persian Pregnancy Apps | 2023 |
| Aydin | Breast cancer-related apps in Google Play and App store: evaluate their functionality and quality | 2023 |
| Baggio | Brazilian Mobile Phone Applications Related to Hand Hygiene and Their Applicability for Healthcare Professionals | 2022 |
| Baptista | A systematic review of smartphone applications and devices for obstructive sleep apnea. | 2022 |
| Barghi | Self-care application for rheumatoid arthritis: Identifying key data elements | 2023 |
| Bearne | Smartphone apps targeting physical activity in people with rheumatoid arthritis: Systematic quality appraisal and content analysis | 2020 |
| Bender | A lot of action, but not in the right direction: Systematic review and content analysis of smartphone applications for the prevention, detection, and management of cancer. | 2013 |
| Bentvelsen | eHealth for the prevention of healthcare-associated infections: a scoping review | 2021 |
| Brown | The Content, Quality, and Behavior Change Techniques in Nutrition-Themed Mobile Apps for Children in Canada: App Review and Evaluation Study | 2022 |
| Brzan | Mobile Applications for Control and Self Management of Diabetes: A Systematic Review | 2016 |
| Cao | Description of apps targeting stroke patients: A review of apps store | 2023 |
| Carvalho | Mobile health technologies for the management of spine disorders: A systematic review of mHealth applications in Brazil | 2022 |
| Chapman | Smartphone apps about crystal methamphetamine (“Ice”): Systematic search in app stores and assessment of composition and quality | 2018 |
| Charalambous | Tools for app- And web-based self-testing of cognitive impairment: Systematic search and evaluation | 2020 |
| Chen | Personalized and Self-Management: Systematic Search and Evaluation Quality Factors and User Preference of Drug Reference Apps in Taiwan | 2021 |
| Chen | Mobile apps for dental caries prevention: Systematic search and quality evaluation | 2021 |
| Cheng | Content and Quality of Infant Feeding Smartphone Apps: Five-Year Update on a Systematic Search and Evaluation | 2020 |
| Cho | Mobile Apps to Improve Brace-Wearing Compliance in Patients with Idiopathic Scoliosis: A Quality Analysis, Functionality Review and Future Directions | 2023 |
| Coe-O'Brien | Outcome measures used in the smartphone applications for the management of low back pain: a systematic scoping review | 2020 |
| Collado-Borrell | Features and functionalities of smartphone apps related to COVID-19: Systematic search in app stores and content analysis | 2020 |
| Coulon | A Systematic, Multi-domain Review of Mobile Smartphone Apps for Evidence-Based Stress Management | 2016 |
| Coulon | A systematic, multi-domain review of mobile smartphone apps for evidence-based stress management | 2016 |
| Cozad | Mobile Health Apps for Patient-Centered Care: Review of United States Rheumatoid Arthritis Apps for Engagement and Activation | 2022 |
| da Silva | Quality assessment of mobile applications on postpartum hemorrhage management | 2023 |
| Dantas | Mobile health technologies for the management of systemic lupus erythematosus: a systematic review | 2020 |
| Dantas | Mobile health technologies for the management of rheumatic diseases: a systematic review of online stores in Brazil | 2021 |
| Dantas | Mobile health technologies for the management of urinary incontinence: A systematic review of online stores in Brazil | 2021 |
| Dantas | Mobile health technologies for the monitoring of menstrual cycle: A systematic review of online stores in Brazil | 2022 |
| Davalbhakta | A Systematic Review of Smartphone Applications Available for Corona Virus Disease 2019 (COVID19) and the Assessment of their Quality Using the Mobile Application Rating Scale (MARS) | 2020 |
| Devan | Evaluation of self-management support functions in apps for people with persistent pain: systematic review | 2019 |
| Emerson | A Systematic Review and Exploration of Smartphone App Interventions for Perinatal Depression With Case Study | 2023 |
| Esmaeeli | A review and content analysis of hemophilia applications: Mobile Application Rating Scale (MARS) Approach | 2023 |
| Evans | Review of Mobile Apps for Women With Anxiety in Pregnancy: Maternity Care Professionals' Guide to Locating and Assessing Anxiety Apps | 2022 |
| Fan | The function and quality of individual epidemic prevention and control apps during the COVID-19 pandemic: A systematic review of Chinese apps | 2022 |
| Feldman | A systematic review of mHealth application interventions for peripartum mood disorders: Trends and evidence in academia and industry. | 2021 |
| Fijačko | The Effects of Gamification and Oral Self-Care on Oral Hygiene in Children: Systematic Search in App Stores and Evaluation of Apps | 2020 |
| Fijačko | Evaluating Quality, Usability, Evidence-Based Content, and Gamification Features in Mobile Learning Apps Designed to Teach Children Basic Life Support: Systematic Search in App Stores and Content Analysis | 2021 |
| Fijaƒçko | A Review of Mortality Risk Prediction Models in Smartphone Applications. | 2021 |
| García-Sánchez | Mobile Health Apps Providing Information on Drugs for Adult Emergency Care: Systematic Search on App Stores and Content Analysis | 2022 |
| Gasteiger | Sticky apps, not sticky hands: A systematic review and content synthesis of hand hygiene mobile apps | 2021 |
| Gasteiger | Patient-facing genetic and genomic mobile apps in the UK: a systematic review of content, functionality, and quality | 2022 |
| Geng | The Most Popular Commercial Weight Management Apps in the Chinese App Store: Analysis of Quality, Features, and Behavior Change Techniques | 2023 |
| Gladman | Measuring the Quality of Clinical Skills Mobile Apps for Student Learning: Systematic Search, Analysis, and Comparison of Two Measurement Scales | 2021 |
| Gong | Quality, functionality, and features of Chinese mobile apps for diabetes self-management: systematic search and evaluation of mobile apps | 2020 |
| Gould | A Systematic Assessment of the Quality of Smartphone Applications for Gastroesophageal Reflux Disease | 2023 |
| Grainger | Apps for people with rheumatoid arthritis to monitor their disease activity: A review of apps for best practice and quality | 2017 |
| Gutierrez | Smartphone Apps for Patients With Hematologic Malignancies: Systematic Review and Evaluation of Content | 2022 |
| Gohari | Drug poisoning management using smartphones: An apps review study based on use case classification model | 2020 |
| Hammond | Psychoeducational Social Anxiety Mobile Apps: Systematic Search in App Stores, Content Analysis, and Evaluation | 2021 |
| Hatem | Mobile Apps for People With Rare Diseases: Review and Quality Assessment Using Mobile App Rating Scale | 2022 |
| Hayman | Quality, features, and presence of behavior change techniques in mobile apps designed to improve physical activity in pregnant women: Systematic search and content analysis | 2021 |
| Hayman | Evaluating Evidence-Based Content, Features of Exercise Instruction, and Expert Involvement in Physical Activity Apps for Pregnant Women: Systematic Search and Content Analysis | 2022 |
| Heales | An evidence-based evaluation of mobile health apps for the management of individuals with lateral elbow tendinopathy using a systematic review framework. | 2021 |
| Ho | Rating of Pelvic Floor Muscle Training Mobile Applications for Treatment of Urinary Incontinence in Women | 2021 |
| Ho | Apps for Promoting Children’s Oral Health: Systematic Search in App Stores and Quality Evaluation | 2022 |
| Hutcherson | Evaluation of Mobile Applications Intended to Aid in Conception Using a Systematic Review Framework | 2020 |
| Jacoby | Systematic Review of Mobile Phone Apps Currently Available to Norwegian Users to Support Diabetes Self-management | 2019 |
| Jannati | Review and content analysis of mobile apps for inflammatory bowel disease management using the mobile application rating scale (MARS): Systematic search in app stores | 2023 |
| Janssen | Mobile applications in radiation oncology-current choices and future potentials | 2023 |
| Jiang | Systematic Review of Colorectal Cancer Screening-Related Apps | 2023 |
| Kapoor | Mobile applications for breast cancer survivorship and self-management: A systematic review | 2020 |
| Karasneh | Smartphone Applications for Period Tracking: Rating and Behavioral Change among Women Users | 2020 |
| Kim | Consumer mobile apps for potential drug-drug interaction check: Systematic review and content analysis using the mobile app rating scale (MARS) | 2018 |
| Kim | A Systematic Review of Korea’s Medication Management Mobile Application | 2023 |
| Koldeweij | CE Accreditation and Barriers to CE Marking of Pediatric Drug Calculators for Mobile Devices: Scoping Review and Qualitative Analysis | 2021 |
| Koumpouros | Pain Management Mobile Applications: A Systematic Review of Commercial and Research Efforts | 2023 |
| Kuo | Understanding the Technological Landscape of Home Health Aides: Scoping Literature Review and a Landscape Analysis of Existing mHealth Apps | 2022 |
| Kuo | A Survey of Mobile Apps for the Care Management of Patients with Dementia | 2022 |
| Kwan | Evaluation of mobile apps targeted at patients with spondyloarthritis for disease monitoring: Systematic app search | 2019 |
| Lalloo | Commercially available smartphone apps to support postoperative pain self-management: Scoping review | 2017 |
| Lancaster | Sleep mHealth Applications and Behavior Change Techniques Evaluation | 2023 |
| Larco | Review and Evaluation of Special Education iOS Apps Using MARS | 2018 |
| Larco | IOS apps for people with intellectual disability: A quality assessment | 2018 |
| Larsen | A Systematic Assessment of Smartphone Tools for Suicide Prevention. | 2016 |
| Lazarevic | Pregnancy Apps for Self-Monitoring: Scoping Review of the Most Popular Global Apps Available in Australia | 2023 |
| Lee | Mobile Apps Leveraged in the COVID-19 Pandemic in East and South-East Asia: Review and Content Analysis | 2021 |
| Lee | Systematic Search and Qualitative Evaluation of Dietary Supplement Mobile Applications: Using the Mobile Application Rating Scale (MARS) | 2023 |
| Lee | Smartphone apps for point-of-care information summaries: Systematic assessment of the quality and content | 2023 |
| Lemes | Mobile Technologies for Monitoring Sleep Time: A Systematic Search of App Stores in Brazil | 2024 |
| Li | Nutrition-Related Mobile Apps in the China App Store: Assessment of Functionality and Quality | 2019 |
| Lopez | Improved readability and functions needed for mHealth apps targeting patients with heart failure: An app store review | 2021 |
| Lorca-Cabrera | Mobile Applications for Caregivers of Individuals with Chronic Conditions and/or Diseases: Quantitative Content Analysis | 2021 |
| Machado | Smartphone apps for the self-management of low back pain: A systematic review | 2016 |
| MacPherson | Do pain management apps use evidence-based psychological components? A systematic review of app content and quality | 2022 |
| Mandelbaum | WhatsApp with the Evidence Base for Behavioral Parent Training Apps? A Systematic Review of Mobile Phone Applications | 2024 |
| Martín-Martín | Evaluation of Android and Apple Store Depression Applications Based on Mobile Application Rating Scale | 2021 |
| Martinengo | Suicide prevention and depression apps’ suicide risk assessment and management: a systematic assessment of adherence to clinical guidelines | 2019 |
| Martinengo | Education on Depression in Mental Health Apps: Systematic Assessment of Characteristics and Adherence to Evidence-Based Guidelines | 2022 |
| Martinez-Millana | App features for type 1 diabetes support and patient empowerment: Systematic literature review and benchmark comparison | 2018 |
| Mauch | Mobile Apps to Support Healthy Family Food Provision: Systematic Assessment of Popular, Commercially Available Apps | 2018 |
| McGarrigle | Map the apps: a rapid review of digital approaches to support the engagement of older adults in strength and balance exercises | 2020 |
| Mehdi | Smartphone and Mobile Health Apps for Tinnitus: Systematic Identification, Analysis, and Assessment | 2020 |
| Mehdi | Smartphone apps in the context of tinnitus: Systematic review | 2020 |
| Messner | Mobile Apps for the Management of Gastrointestinal Diseases: Systematic Search and Evaluation Within App Stores | 2022 |
| Metelmann | Medical correctness and user friendliness of available apps for cardiopulmonary resuscitation: Systematic search combined with guideline adherence and usability evaluation | 2018 |
| Mieso | Mobile Phone Applications to Support Breastfeeding Among African-American Women: a Scoping Review | 2022 |
| Mourão | Aplicativos móveis para promoção de cuidados com pé diabético: revisão de escopo. (Mobile applications to promote diabetic foot care: scoping review) | 2022 |
| Muntaner-Mas | A Systematic Review of Fitness Apps and Their Potential Clinical and Sports Utility for Objective and Remote Assessment of Cardiorespiratory Fitness. | 2019 |
| Musgrave | Mobile phone apps in Australia for improving pregnancy outcomes: systematic search on app stores | 2020 |
| Myers | Evaluating Commercially Available Mobile Apps for Depression Self-Management | 2020 |
| Napolitano | Erectile dysfunction and mobile phone applications: Quality, content and adherence to European Association guidelines on male sexual dysfunction | 2022 |
| Napolitano | Premature ejaculation in the era of mobile health application: A current analysis and evaluation of adherence to EAU guidelines | 2022 |
| Narrillos-Moraza | Mobile Apps for Hematological Conditions: Review and Content Analysis Using the Mobile App Rating Scale | 2022 |
| Noronha | Mobile Applications for COVID-19: A Scoping Review of the Initial Response in Canada | 2020 |
| Nuamah | Technologies for opioid use disorder management: Mobile app search and scoping review | 2020 |
| O'Connor | A review of the quality and content of mobile apps to support lifestyle modifications following a transient ischaemic attack or 'minor' stroke. | 2021 |
| Ouhbi | Free Blood Donation Mobile Applications | 2015 |
| Pacheco | Clinical wisdom in the age of computer apps: A systematic review of four functions that may complement clinical treatment | 2022 |
| Paganini | Stress Management Apps: Systematic Search and Multidimensional Assessment of Quality and Characteristics | 2023 |
| Paripoorani | A systematic review of menopause apps with an emphasis on osteoporosis | 2023 |
| Păsărelu | Attention-deficit/ hyperactivity disorder mobile apps: A systematic review | 2020 |
| Pearsons | Atrial fibrillation self-management: A mobile telephone app scoping review and content analysis | 2021 |
| Pichon | The messiness of the menstruator: assessing personas and functionalities of menstrual tracking apps | 2022 |
| Piran | Medical Mobile Applications for Stroke Survivors and Caregivers | 2019 |
| Portenhauser | Mobile Apps for Older Adults: Systematic Search and Evaluation Within Online Stores | 2021 |
| Qari | Assessing the quality of mobile apps for oral health: content analysis and usability | 2019 |
| Queiroz | mHealth strategies related to HIV postexposure prophylaxis knowledge and access: Systematic literature review, technology prospecting of patent databases, and systematic search on app stores | 2021 |
| Richardson | Evaluation of mobile apps targeted to parents of infants in the neonatal intensive care unit: Systematic app review | 2019 |
| Rickard | Systematic assessment of the quality and integrity of popular mental health smartphone apps using the American Psychiatric Association's app evaluation model | 2022 |
| Robinson | Asthma mHealth apps: Their quality and consistency with international guidelines | 2023 |
| Robinson | Functionality and Quality of Asthma mHealth Apps and Their Consistency With International Guidelines: Structured Search and Evaluation. | 2024 |
| Romero-Jimenez | The Characteristics and Functionalities of Mobile Apps Aimed at Patients Diagnosed With Immune-Mediated Inflammatory Diseases: Systematic App Search | 2022 |
| Roncero | Mobile health apps for medical emergencies: Systematic review | 2020 |
| Ruggiano | Chatbots to support people with dementia and their caregivers: Systematic review of functions and quality | 2021 |
| Russell | A Systematic Review and Qualitative Analysis of Existing Dietary Mobile Applications for People With Chronic Kidney Disease | 2022 |
| Samad | Smartphone apps for tracking food consumption and recommendations: Evaluating artificial intelligence-based functionalities, features and quality of current apps | 2022 |
| Sardi | Mobile health applications for postnatal care: Review and analysis of functionalities and technical features | 2020 |
| Scantling-Birch | A Review of Smartphone Apps Used for Toric Intraocular Lens Calculation and Alignment. | 2022 |
| Schmeelk | Monitoring Symptoms of COVID-19: Review of Mobile Apps | 2022 |
| Schoeppe | Apps to improve diet, physical activity and sedentary behaviour in children and adolescents: A review of quality, features and behaviour change techniques | 2017 |
| Shen | Mobile apps for drug–Drug interaction checks in Chinese app stores: Systematic review and content analysis | 2021 |
| Siqueira do Prado | Behavior Change Content, Understandability, and Actionability of Chronic Condition Self-Management Apps Available in France: Systematic Search and Evaluation | 2019 |
| Song | Evaluating Chinese Mobile Health Apps for Ankylosing Spondylitis Management: Systematic App Search | 2021 |
| Soto-Bagaria | Mobile applications to prescribe physical exercise in frail older adults: review of the available tools in app stores. | 2023 |
| Spadaro | mHealth Solutions for Perinatal Mental Health: Scoping Review and Appraisal Following the mHealth Index and Navigation Database Framework | 2022 |
| Stifani | There's an App for it: A systematic review of mobile apps providing information about abortion using a revised MARS scale. | 2023 |
| Sucala | Hypnosis-there's an app for that: A systematic review of hypnosis apps | 2013 |
| Sujarwoto | COVID-19 Mobile Health Apps: An Overview of Mobile Applications in Indonesia | 2022 |
| Sunjaya | Efficacy of self-management mobile applications for patients with breathlessness: Systematic review and quality assessment of publicly available applications | 2022 |
| Tavares | Mobile applications for training plan using android devices: A systematic review and a taxonomy proposal | 2020 |
| Thomas | Empowerment through technology: A systematic evaluation of the content and quality of mobile applications to empower individuals with cancer | 2022 |
| Trecca | Mobile Applications in Otolaryngology: A Systematic Review of the Literature, Apple App Store and the Google Play Store | 2021 |
| Trépanier | Smartphone apps for menstrual pain and symptom management: A scoping review | 2023 |
| Tsai | Evaluating the effectiveness and quality of mobile applications for perinatal depression and anxiety: A systematic review and meta-analysis | 2022 |
| Tucker | Comprehensively addressing postpartum maternal health: a content and image review of commercially available mobile health apps | 2021 |
| Vaezipour | Mobile apps for speech-language therapy in adults with communication disorders: Review of content and quality | 2020 |
| Vaitkienƒó | Digital Health Solutions for Chronic Illnesses: A Systematic Review of Mobile Health Apps and Quality Analysis with Mobile App Rating Scale. | 2022 |
| Vandersman | Is There an App for That? A Scoping Review of Apps for Care Workers in the Aged Care Sector | 2021 |
| Venugopal | A systematic review of smartphone apps for gastro-oesophageal reflux disease: The need for regulation and medical professional involvement | 2021 |
| Vercell | Patient-facing cancer mobile apps that enable patient reported outcome data to be collected: A systematic review of content, functionality, quality, and ability to integrate with electronic health records | 2023 |
| Villasana | Mobile applications for the promotion and support of healthy nutrition and physical activity habits: A systematic review, extraction of features and taxonomy proposal | 2019 |
| Wasil | A review of popular smartphone apps for depression and anxiety: Assessing the inclusion of evidence-based content | 2019 |
| Wasil | Smartphone apps for eating disorders: A systematic review of evidence-based content and application of user-adjusted analyses | 2021 |
| Wasserman | An Evaluation of Interactive mHealth Applications for Adults Living with Cancer | 2023 |
| Wiemker | Digital Assessment Tools Using Animation Features to Quantify Alcohol Consumption: Systematic App Store and Literature Review | 2022 |
| Winoker | Systematic Evaluation of Smartphone Applications for the Medical Management of Nephrolithiasis | 2021 |
| Zarnowiecki | A systematic evaluation of digital nutrition promotion websites and apps for supporting parents to influence children's nutrition. | 2020 |
| Zi | A systematic review of mindfulness mobile apps: considering content quality and user engagement elements | 2021 |

**Dot density map displaying the locations of the app market searches, when clearly named (n=85).**


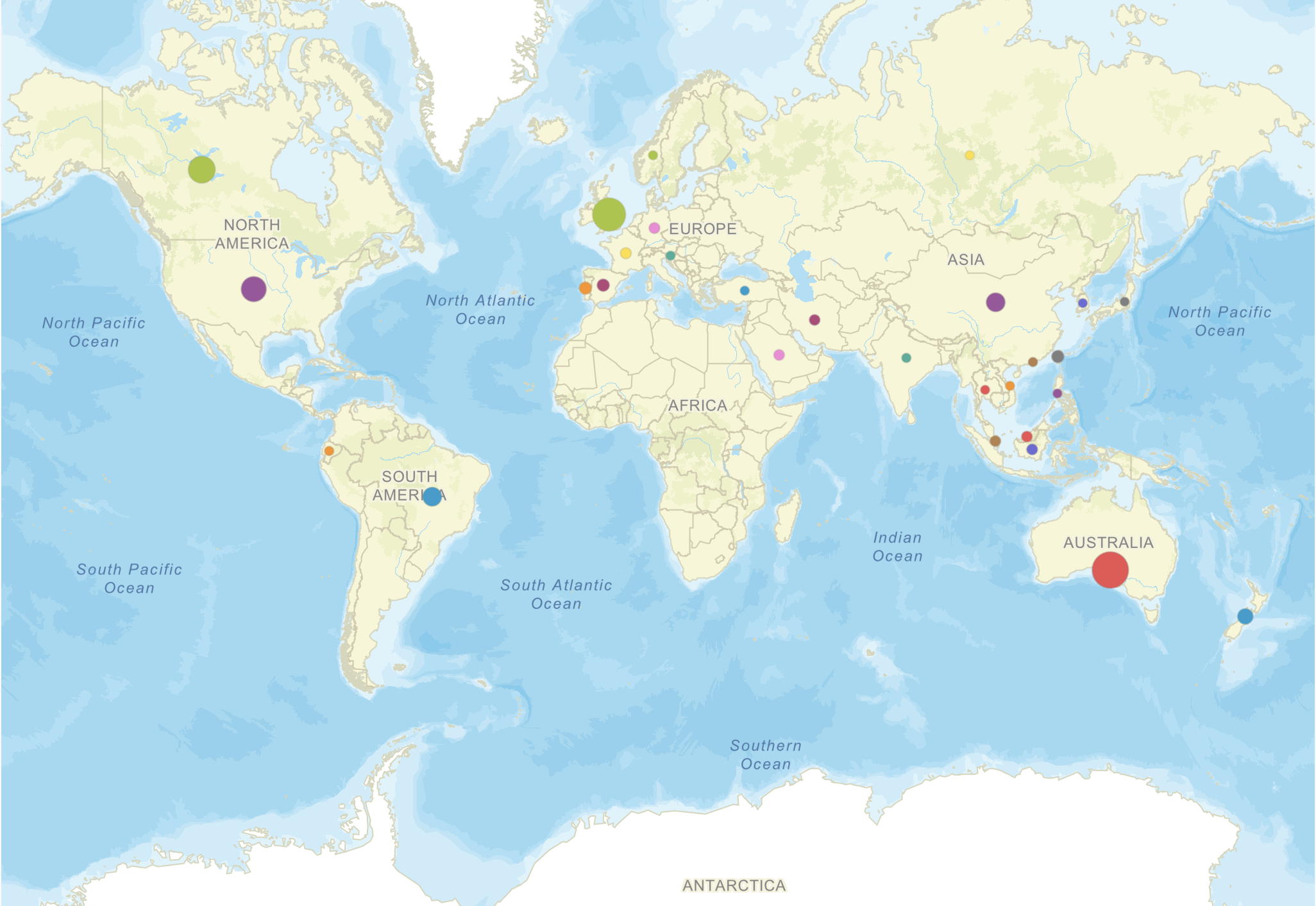


Note: larger dots indicate a higher number (greater density) of reviews.

**Commonly used existing (including validated) measures, frameworks and their sources.**

| **OUTCOMES/ MEASURES/ FRAMEWORKS** | **SOURCES** |
| --- | --- |
| **Quality** | |
| Mobile App Rating Scale (MARS)  -User version (u-MARS)  -German version (MARS-G)  -Korean version (MARS-K) | Stoyanov, S. R., Hides, L., Kavanagh, D. J., Zelenko, O., Tjondronegoro, D., & Mani, M. (2015). Mobile app rating scale: a new tool for assessing the quality of health mobile apps. *JMIR mHealth and uHealth*, *3*(1), e27. <https://doi.org/10.2196/mhealth.3422>  Stoyanov SR, Hides L, Kavanagh DJ & Wilson H. (2016). Development and Validation of the User Version of the Mobile Application Rating Scale (uMARS) *JMIR Mhealth Uhealth, 4*(2):e72 doi: [10.2196/mhealth.5849](https://doi.org/10.2196/mhealth.5849)  Messner, E. M., Terhorst, Y., Barke, A., Baumeister, H., Stoyanov, S., Hides, L., Kavanagh, D., Pryss, R., Sander, L., & Probst, T. (2020). The German Version of the Mobile App Rating Scale (MARS-G): Development and Validation Study. *JMIR mHealth and uHealth*, *8*(3), e14479. https://doi.org/10.2196/14479  Hee Ko KK, Kim SK, Lee Y, Lee JY & Stoyanov SR. (2022). Validation of a Korean version of mobile app rating scale (MARS) for apps targeting disease management. *Health Informatics Journal, 28*(2). doi:[10.1177/14604582221091975](https://doi.org/10.1177/14604582221091975) |
| Quality attributes of chatbots and conversational agents | Radziwill, Nicole & Benton, Morgan. (2017). Evaluating Quality of Chatbots and Intelligent Conversational Agents. <https://www.researchgate.net/publication/316184347_Evaluating_Quality_of_Chatbots_and_Intelligent_Conversational_Agents> |
| APA App Evaluation Model | <https://www.psychiatry.org/psychiatrists/practice/mental-health-apps/the-app-evaluation-model> |
| Health-Related Website Evaluation Form | <https://www.carlbring.se/form/itform_eng.pdf> |
| A New Tool for Nutrition App Quality Evaluation (AQEL) | DiFilippo KN, Huang W & Chapman-Novakofski KM. (2017). A New Tool for Nutrition App Quality Evaluation (AQEL): Development, Validation, and Reliability Testing. *JMIR Mhealth Uhealth, 5*(10):e163 doi: [10.2196/mhealth.7441](https://doi.org/10.2196/mhealth.7441) |
| THESIS | Levine, D. M., Co, Z., Newmark, L. P., Groisser, A. R., Holmgren, A. J., Haas, J. S., & Bates, D. W. (2020). Design and testing of a mobile health application rating tool. *NPJ digital medicine*, *3*, 74. https://doi.org/10.1038/s41746-020-0268-9 |
| Johns Hopkins Digital Health Scorecard | Sedhom, R., McShea, M.J., Cohen, A.B. *et al.* (2021). Mobile app validation: a digital health scorecard approach. *npj Digit. Med.* 4, 111. https://doi.org/10.1038/s41746-021-00476-7 |
| Suitability Assessment of Materials (SAM) tool | <https://aspiruslibrary.org/literacy/sam.pdf> |
| Silberg Scale | Silberg, W. M., Lundberg, G. D., & Musacchio, R. A. (1997). Assessing, controlling, and assuring the quality of medical information on the Internet: Caveant lector et viewor—Let the reader and viewer beware. *JAMA, 277*(15), 1244-1245. https://doi.org/10.1001/jama.1997.03540390074039 |
| **Functionality** | |
| IMS Institute for Healthcare Informatics functionality score | <http://ignacioriesgo.es/wp-content/uploads/2014/03/iihi_patient_apps_report_editora_39_2_1.pdf> |
| A New Tool for Nutrition App Quality Evaluation (AQEL) | DiFilippo KN, Huang W & Chapman-Novakofski KM. (2017). A New Tool for Nutrition App Quality Evaluation (AQEL): Development, Validation, and Reliability Testing. *JMIR Mhealth Uhealth, 5*(10):e163 doi: [10.2196/mhealth.7441](https://doi.org/10.2196/mhealth.7441) |
| Quality attributes of chatbots and conversational agents | Radziwill, Nicole & Benton, Morgan. (2017). Evaluating Quality of Chatbots and Intelligent Conversational Agents. <https://www.researchgate.net/publication/316184347_Evaluating_Quality_of_Chatbots_and_Intelligent_Conversational_Agents> |
| MARS (functionality dimension) | Stoyanov, S. R., Hides, L., Kavanagh, D. J., Zelenko, O., Tjondronegoro, D., & Mani, M. (2015). Mobile app rating scale: a new tool for assessing the quality of health mobile apps. *JMIR mHealth and uHealth*, *3*(1), e27. <https://doi.org/10.2196/mhealth.3422>  See also uMARS, MARS-G and MARS-K (cited above) |
| **Usability** | |
| SUS (System Usability Scale) | Brooke, J. (1996). *SUS: A 'quick and dirty' usability scale*. In P. W. Jordan, B. Thomas, B. A. Weerdmeester, & I. L. McClelland (Eds.), Usability evaluation in industry (pp. 189-194). Taylor & Francis. |
| MARS (Item 7- ease of use) | Stoyanov, S. R., Hides, L., Kavanagh, D. J., Zelenko, O., Tjondronegoro, D., & Mani, M. (2015). Mobile app rating scale: a new tool for assessing the quality of health mobile apps. *JMIR mHealth and uHealth*, *3*(1), e27. <https://doi.org/10.2196/mhealth.3422>  See also uMARS, MARS-G and MARS-K (cited above) |
| THESIS | Levine, D. M., Co, Z., Newmark, L. P., Groisser, A. R., Holmgren, A. J., Haas, J. S., & Bates, D. W. (2020). Design and testing of a mobile health application rating tool. *NPJ digital medicine*, *3*, 74. https://doi.org/10.1038/s41746-020-0268-9 |
| Johns Hopkins Digital Health Scorecard | Sedhom, R., McShea, M.J., Cohen, A.B. *et al.* (2021). Mobile app validation: a digital health scorecard approach. *npj Digit. Med.* 4, 111. https://doi.org/10.1038/s41746-021-00476-7 |
| Quality attributes of chatbots and conversational agents | Radziwill, Nicole & Benton, Morgan. (2017). Evaluating Quality of Chatbots and Intelligent Conversational Agents. <https://www.researchgate.net/publication/316184347_Evaluating_Quality_of_Chatbots_and_Intelligent_Conversational_Agents> |
| MIND Framework | <https://mindapps.org/FrameworkQuestions> |
| Nielson’s Usability Heuristic  Note: This was adapted. | <https://www.nngroup.com/articles/ten-usability-heuristics/> |
| Smartphone Usability questionnaire (SURe) | Wangenheim CG, Borgatto AF, Nunes JV, Lacerda TC, Oliveira RJ, Krone C, et al. (2014). Sure: uma proposta de questionário e escala para avaliar a usabilidade de aplicações para smartphones pós-teste de usabilidade. 6ta. Conferencia Latinoamericana de Diseño de Interacción. http://bibliotecadigital.uca.edu.ar/greenstone/cgi-bin/library. cgi?a=d&c=Ponencias&d=sure-proposta-questionario-escala |
| **Efficacy & evidence base** | |
| MARS (Item 19) | Stoyanov, S. R., Hides, L., Kavanagh, D. J., Zelenko, O., Tjondronegoro, D., & Mani, M. (2015). Mobile app rating scale: a new tool for assessing the quality of health mobile apps. *JMIR mHealth and uHealth*, *3*(1), e27. <https://doi.org/10.2196/mhealth.3422> |
| MIND Framework | <https://mindapps.org/FrameworkQuestions> |
| **Privacy/security** | |
| MIND Framework | <https://mindapps.org/FrameworkQuestions> |
| European Union GDPR | <https://www.consilium.europa.eu/en/policies/data-protection/data-protection-regulation/#:~:text=data%20protection%20rules-,What%20is%20the%20GDPR%3F,the%201995%20data%20protection%20directive> |
| Information Commissioner’s Office in the UK | <https://ico.org.uk/> |
| Online Trust Alliance | <https://www.internetsociety.org/ota/> |
| Health on the Net (HON) Code | <https://cdn.ciussscentreouest.ca/documents/hgj/pfrc/HONcode/HONCode-Overview2022-EN.pdf?16844310#:~:text=Two%20decades%20on%2C%20the%20HONcode,foundation%20and%20non%2D%20governmental%20organization> |
| U.S. Department of Health and Human Services regulations protecting privacy and security of health information | <https://www.hhs.gov/hipaa/for-professionals/privacy/index.html> |
| **Accessibility** | |
| Flesch-Kincaid metrics  -Reading ease score -Grade level (USA equivalent)  The metrics can be generated using Microsoft Word. | Flesch, R. (1979). How to Write Plain English: A Book for Lawyers and Consumers. Harper & Row.  Kincaid, J., Fishburne, R., Rogers, R., & Chissom, B. (1975). Derivation Of New Readability Formulas (Automated Readability Index, Fog Count And Flesch Reading Ease Formula) For Navy Enlisted Personnel. Research Branch Report 8-75.  <https://support.microsoft.com/en-gb/office/get-your-document-s-readability-and-level-statistics-85b4969e-e80a-4777-8dd3-f7fc3c8b3fd2> |
| The Patient Education Materials Assessment Tool for Audio-Visual material (PEMAT- A/V) | <https://www.ahrq.gov/health-literacy/patient-education/pemat-av.html> |
| MIND Framework | <https://mindapps.org/FrameworkQuestions> |
| **Other measures/outcomes** | |
| Behaviour Change Techniques (BCTs) | Michie S, Richardson M, Johnston M, Abraham C, Francis J, Hardeman W, Eccles MP, Cane J & Wood CE. (2013). The behavior change technique taxonomy (v1) of 93 hierarchically clustered techniques: building an international consensus for the reporting of behavior change interventions. *Ann Behav Med, 46*(1):81-95. doi: 10.1007/s12160-013-9486-6.  Michie S, Ashford S, Sniehotta FF, Dombrowski SU, Bishop A & French DP. (2011). A refined taxonomy of behaviour change techniques to help people change their physical activity and healthy eating behaviours: the CALO-RE taxonomy. *Psychol Health, 26*(11):1479-98. doi: 10.1080/08870446.2010.540664. |
| ABACUS tool | McKay FH, Slykerman S & Dunn M. (2019). The App Behavior Change Scale: Creation of a Scale to Assess the Potential of Apps to Promote Behavior Change. *JMIR Mhealth Uhealth, 7(*1):e11130. doi: 10.2196/11130. |
| ORCHA | <https://orchahealth.com/> |
| Health-Related Website Evaluation Form | <https://www.carlbring.se/form/itform_eng.pdf> |
| PsyberGuide credibility scores | https://onemindpsyberguide.org/guide/about-psyberguide/#:~:text=The%20Credibility%20Score%20combines%20information,specifically%20for%20the%20tool%20itself |
| Trust It or Trash It | <https://trustortrash.org/> |
| Mobile App Rubric for Learning (MARuL) | Gladman T, Tylee G, Gallagher S, Mair J, Rennie SC & Grainger R. (2020). A Tool for Rating the Value of Health Education Mobile Apps to Enhance Student Learning (MARuL): Development and Usability Study. *JMIR Mhealth Uhealth. 31;*8(7):e18015. doi: 10.2196/18015. |
| APA App Evaluation Model | <https://www.psychiatry.org/psychiatrists/practice/mental-health-apps/the-app-evaluation-model> |
| MIND Framework | <https://mindapps.org/FrameworkQuestions> |
| Behaviour Change Techniques (BCTs) | Michie S, Richardson M, Johnston M, Abraham C, Francis J, Hardeman W, Eccles MP, Cane J & Wood CE. (2013). The behavior change technique taxonomy (v1) of 93 hierarchically clustered techniques: building an international consensus for the reporting of behavior change interventions. *Ann Behav Med, 46*(1):81-95. doi: 10.1007/s12160-013-9486-6.  Michie S, Ashford S, Sniehotta FF, Dombrowski SU, Bishop A & French DP. (2011). A refined taxonomy of behaviour change techniques to help people change their physical activity and healthy eating behaviours: the CALO-RE taxonomy. *Psychol Health, 26*(11):1479-98. doi: 10.1080/08870446.2010.540664. |

**Reporting of the PRISMA Abstracts items in the included 171 systematic mHealth app reviews.**

| **PRISMA Abstracts Items** | **Reported number (%)** | **Not reported number (%)** | **Unclear* number (%)** | **Modified number (%)** | **Notes (if applicable)** |
| --- | --- | --- | --- | --- | --- |
| 1. Identify the report as a systematic review. | 101 (59) | 70 (41) | - | - | N/A |
| 2. Provide an explicit statement of the main objective(s) or question(s) the review addresses. | 159 (93) | 12 (7) | - | - | N/A |
| 3. Specify the inclusion and exclusion criteria for the review. | 16 (9) | 109 (64) | 46 (27) | - | 27% failed to report both the inclusion and exclusion criteria of the apps. |
| 4. Specify the information sources (e.g., databases, registers) used to identify studies and the date when each was last searched. | 88 (51) | 25 (15) | 58 (34) | - | Databases (i.e., app stores) and date of searches reported. 34% did not report both aspects. |
| 5. Specify the methods used to assess risk of bias in the included studies. | - | 165 (96) | - | 6 (4) | Risk of bias from evaluating the apps is assessed. Inter-rater reliability of the app evaluation measures assessed using Cohens Kappa or Intraclass Correlation Coefficient. |
| 6. Specify the methods used to present and synthesise results. | 54 (32) | 117 (68) | - | - | N/A |
| 7. Give the total number of included studies and participants and summarise relevant characteristics of studies. | - | 10 (6) | 27 (16) | 134 (78) | Total number of reviewed apps reported and relevant characteristics summarised (e.g., platform available, care focus, language) |
| 8. Present results for main outcomes, preferably indicating the number of included studies and participants for  each. If meta-analysis was done, report the summary estimate and confidence/credible interval. If comparing groups, indicate the direction of the effect (i.e. which group is favoured) | - | 29 (17) | - | 142 (83) | Main outcomes presented for the app evaluations (e.g., statistics for MARS scores, mean number of behaviour change techniques present). |
| 9. Provide a brief summary of the limitations of the evidence included in the review (e.g. study risk of bias, inconsistency and imprecision). | 22 (13) | 149 (87) | - | - | N/A |
| 10. Provide a general interpretation of the results and important implications. | 161 (94) | 10 (6) | - | - | N/A |
| 11. Specify the primary source of funding for the review. | 120 (70) | 51 (30) | - | - | Considered ‘reported’ if anywhere in the publication, due to individual formatting requirements of publications. |
| 12. Provide the register name and registration number. | 12 (7) | 159 (93) | - | - | N/A |

Notes:
*unclear refers to items that are partially reported. **not applicable

Orange highlighting indicates items that were not reported by >85% of the reviews. Green indicates items that had been modified by >30% of the reviews.

**Reporting of the PRISMA items in the included 171 systematic mHealth app reviews.**

| **PRISMA Items** | **Reported number (%)** | **Not reported number (%)** | **Unclear* number (%)** | **Modified number (%)** | **Notes (if applicable)** |
| --- | --- | --- | --- | --- | --- |
| 1. Identify the report as a systematic review. | 101 (59) | 70 (41) | - | - | N/A** |
| 2. See the PRISMA 2020 for Abstracts checklist. | - | - | - | - | See Table 2 |
| 3. Describe the rationale for the review in the context of existing knowledge. | 171 (100) | - | - | - | N/A |
| 4. Provide an explicit statement of the objective(s) or question(s) the review addresses. | 171 (100) | - | - | - | N/A |
| 5. Specify the inclusion and exclusion criteria for the review and how studies were grouped for the syntheses. | 122 (71) | 9 (5) | 40 (23) | - | 23% failed to report both the inclusion and exclusion criteria of the apps. |
| 6. Specify all databases, registers, websites, organisations, reference lists and other sources searched or consulted to identify studies. Specify the date when each source was last searched or consulted. | 152 (89) | 2 (1) | 17 (10) | - | Databases (i.e., app stores) and date of searches reported. 10% did not report both aspects. |
| 7. Present the full search strategies for all databases, registers and websites, including any filters and limits used. | 165 (96) | 3 (2) | 3 (2) | - | N/A |
| 8. Specify the methods used to decide whether a study met the inclusion criteria of the review, including how many reviewers screened each record and each report retrieved, whether they worked independently, and if applicable, details of automation tools used in the process. | 109 (64) | 29 (17) | 33 (19) | - | 19% partially reported the methods of determining whether an app met the inclusion criteria and the screening methods. |
| 9. Specify the methods used to collect data from reports, including how many reviewers collected data from each report, whether they worked independently, any processes for obtaining or confirming data from study investigators, and if applicable, details of automation tools used in the process. | 121 (71) | 19 (11) | 31 (18) | - | 18% partially reported the methods used to collect data from the apps. |
| 10a. List and define all outcomes for which data were sought. Specify whether all results that were compatible with each outcome domain in each study were sought (e.g. for all measures, time points, analyses), and if not, the methods used to decide which results to collect. | 125 (73) | 8 (5) | 38 (22) | - | 22% did not clearly report all the outcomes for which data were sought. |
| 10b. List and define all other variables for which data were sought (e.g., participant and intervention characteristics, funding sources). Describe any assumptions made about any missing or unclear information. | - | 171 (100) | - | - | N/A |
| 11. Specify the methods used to assess risk of bias in the included studies, including details of the tool(s) used, how many reviewers assessed each study and whether they worked independently, and if applicable, details of automation tools used in the process. | - | 104 (61) | - | 67 (39) | Modified to assess risk of bias from evaluating the apps. This included inter-rater reliability of the app evaluation measures (e.g., MARS) using Cohens Kappa or Intraclass Correlation Coefficient. |
| 12. Specify for each outcome the effect measure(s) (e.g., risk ratio, mean difference) used in the synthesis or presentation of results. | 3 (2) | 166 (97) | - | 2 (1) | N/A |
| 13a. Describe the processes used to decide which studies were eligible for each synthesis (e.g. tabulating the study intervention characteristics and comparing against the planned groups for each synthesis (item #5)). | 1 (1) | 166 (97) | - | 4 (2) | N/A |
| 13b. Describe any methods required to prepare the data for presentation or synthesis, such as handling of missing summary statistics, or data conversions. | 2 (1) | 169 (99) | - | - | N/A |
| 13c. Describe any methods used to tabulate or visually display results of individual studies and syntheses. | 3 (2) | 168 (98) | - | - | N/A |
| 13d. Describe any methods used to synthesize results and provide a rationale for the choice(s). If meta-analysis was performed, describe the model(s), method(s) to identify the presence and extent of statistical heterogeneity, and software package(s) used. | 128 (75) | 43 (25) | - | - | N/A |
| 13e. Describe any methods used to explore possible causes of heterogeneity among study results (e.g., subgroup analysis, meta-regression). | - | 170 (99) | - | 1 (1) | 1 review conducted a subgroup analysis to assess whether the number of app downloads was associated with the educational content, quality or number of features. |
| 13f. Describe any sensitivity analyses conducted to assess robustness of the synthesized results. | - | 171 (100) | - | - | N/A |
| 14. Describe any methods used to assess risk of bias due to missing results in a synthesis (arising from reporting biases). | - | 171 (100) | - | - | N/A |
| 15. Describe any methods used to assess certainty (or confidence) in the body of evidence for an outcome. | - | 171 (100) | - | - | N/A |
| 16a. Describe the results of the search and selection process, from the number of records identified in the search to the number of studies included in the review, ideally using a flow diagram. | 170 (99) | 1 (1) | - | - | N/A |
| 16b. Cite studies that might appear to meet the inclusion criteria, but which were excluded, and explain why they were excluded. | - | 163 (95) | - | 8 (5) | 5% of reviews named the apps that had been screened but were excluded. This information was often in the supplementary file. |
| 17. Cite each included study and present its characteristics. | - | 29 (17) | - | 142 (83) | Reviewed apps were named in-text or in the supplementary file. Key characteristics presented (see Figure 4). Note: version and developer were not always named. |
| 18. Present assessments of risk of bias for each included study. | - | 117 (68) | - | 54 (32) | 32% presented inter-rater reliability from app evaluators e.g., Cohens Kappa, Intraclass Correlation Coefficient, Kendall’s coefficient of concordance or raw agreement (%). |
| 19. For all outcomes, present, for each study: (a) summary statistics for each group (where appropriate) and (b) an effect estimate and its precision (e.g. confidence/credible interval), ideally using structured tables or plots. | 2 (1) | 169 (99) | - | - | N/A |
| 20a. For each synthesis, briefly summarise the characteristics and risk of bias among contributing studies. | - | 171 (100) | - | - | N/A |
| 20b. Present results of all statistical syntheses conducted. If meta-analysis was done, present for each the summary estimate and its precision (e.g. confidence/credible interval) and measures of statistical heterogeneity. If comparing groups, describe the direction of the effect. | 148 (87) | 23 (13) | - | - | 87% provided results of statistical analysis including descriptive or inferential statistics. Meta-analysis was not relevant. |
| 20c. Present results of all investigations of possible causes of heterogeneity among study results. |  | 168 (98) | - | 3 (2) | Example of modification includes subgroup analysis to assess whether the number of app downloads was associated with the educational content, quality or number of features. |
| 20d. Present results of all sensitivity analyses conducted to assess the robustness of the synthesized results. | 1 (1) | 170 (99) | - | - | N/A |
| 21. Present assessments of risk of bias due to missing results (arising from reporting biases) for each synthesis assessed. | - | 171 (100) | - | - | N/A |
| 22. Present assessments of certainty (or confidence) in the body of evidence for each outcome assessed. | - | 171 (100) | - | - | N/A |
| 23a. Provide a general interpretation of the results in the context of other evidence. | 169 (99) | 2 (1) | - | - | N/A |
| 23b. Discuss any limitations of the evidence included in the review. | - | 50 (29) | - | 121 (71) | Discussed limitations of the apps reviewed. |
| 23c. Discuss any limitations of the review processes used. | 149 (87) | 22 (13) | - | - | N/A |
| 23d. Discuss implications of the results for practice, policy, and future research. | 156 (91) | 15 (9) | - | - | Also discussed future technology development and implications for app review (research) methods. |
| 24a. Provide registration information for the review, including register name and registration number, or state that the review was not registered. | 18 (11) | 152 (89) | 1 (1) | - | 1 review provided registration details for the literature review component, not the app review. |
| 24b. Indicate where the review protocol can be accessed, or state that a protocol was not prepared. | 17 (10) | 153 (89) | 1 (1) | - | N/A |
| 24c. Describe and explain any amendments to information provided at registration or in the protocol. | - | 171 (100) | - | - | N/A |
| 25. Describe sources of financial or non-financial support for the review, and the role of the funders or sponsors in the review. | 119 (70) | 52 (30) | - | - | N/A |
| 26. Declare any competing interests of review authors. | 150 (88) | 21 (12) | - | - | N/A |
| 27. Report which of the following are publicly available and where they can be found: template data collection forms; data extracted from included studies; data used for all analyses; analytic code; any other materials used in the review. | 74 (43) | 97 (57) | - | - | N/A |

Notes:

*unclear refers to items that are partially reported. **not applicable

Orange highlighting indicates items that were not reported by >85% of the reviews. Green indicates items that had been modified by >30% of the reviews.
